# Nine new species of black lichenicolous fungi from the genus *Cladophialophora* (Chaetothyriales) from two different climatic zones of China

**DOI:** 10.3389/fmicb.2023.1191818

**Published:** 2023-06-16

**Authors:** Runlei Chang, Yichen Wang, Yanyu Liu, Yiran Wang, Shiguo Li, Guoyan Zhao, Susu Zhang, Meixue Dai, Xiaoxiao Zheng, Tanay Bose, Hongli Si

**Affiliations:** ^1^College of Life Sciences, Shandong Normal University, Jinan, China; ^2^Dongying Institute, Shandong Normal University, Dongying, China; ^3^Department of Biochemistry, Genetics and Microbiology, Forestry and Agricultural Biotechnology Institute (FABI), University of Pretoria, Pretoria, South Africa

**Keywords:** Ascomycota, biodiversity, Chaetothyriomycetidae, lichens, multi-gene phylogeny, Yunnan Province, Inner Mongolia Autonomous Region

## Abstract

Lichenicolous fungi are parasites of lichens. Many of these fungi are referred to as “black fungi”. A diversity of these black fungi include species that are pathogenic to humans and plants. A majority of black fungi reside in the phylum Ascomycota within the sub-classes Chaetothyriomycetidae and Dothideomycetidae. To explore the diversity of lichenicolous “black fungi” associated with lichens in China, we conducted several field surveys in the Inner Mongolia Autonomous Region and Yunnan Province between 2019 and 2020. We recovered 1,587 fungal isolates from the lichens collected during these surveys. During the preliminary identification of these isolates using the complete internal transcribed spacer (ITS), partial large subunit of nuclear ribosomal RNA gene (LSU), and small subunit of nuclear ribosomal RNA gene (SSU), we identified 15 fungal isolates from the genus *Cladophialophora*. However, these isolates had low sequence similarities with all known species from the genus. Therefore, we amplified additional gene regions, such as, translation elongation factor (TEF) and partial β-tubulin gene (TUB), and constructed a multi-gene phylogeny using maximum likelihood, maximum parsimony, and Bayesian inference. In our datasets, we included type sequences where available for all *Cladophialophora* species. Phylogenetic analyses revealed that none of the 15 isolates belonged to any of the previously described species in the genus. Therefore, using both morphological and molecular data, we classified these 15 isolates as nine new species within the genus *Cladophialophora*: *C. flavoparmeliae*, *C. guttulate*, *C. heterodermiae*, *C. holosericea*, *C. lichenis, C. moniliformis*, *C. mongoliae*, *C. olivacea*, and *C. yunnanensis*. The outcome from this study shows that lichens are an important refugia for black lichenicolous fungi, such as those from Chaetothyriales.

## 1. Introduction

“Black fungi” are characterized by dark-colored mycelia due to the buildup of melanin in their cell walls (Ametrano et al., 2019). This melanization of the cell wall allows them to colonize extreme habitats, such as rock surfaces and lichen thalli (Gostinčar et al., 2012). These groups of fungi can also have an assortment of thallus morphologies, such as yeast, pseudo-filamentous, and filamentous (Gostinčar et al., 2012). When co-cultured with compatible algae, several black fungi show an earlier stage of lichenization, such as the formation of lichen-like fungal plectenchyme (Brunauer et al., 2007; Muggia and Grube, 2018). A majority of black fungi reside in the phylum Ascomycota within the sub-classes Chaetothyriomycetidae and Dothideomycetidae (Harutyunyan et al., 2008). One such genus is *Cladophialophora*, from the order Chaetothyriales.

*Cladophialophora* is a genus of asexual dematiaceous (darkly pigmented) fungi. This genus of fungi is distinguished by branched or unbranched chains of conidia with hyaline conidial scars produced through blastic conidiogenesis (Sutton et al., 2009; Obase et al., 2016; Khan and Sohnle, 2019). Currently, *Cladophialophora* includes about 45 accepted species with diverse ecological habitats. Species of *Cladophialophora* has been isolated from an assortment of substrates, such as plants, soil, sports drinks, human, and lichens (Obase et al., 2016; Usui et al., 2016; Tang et al., 2017). At least 11 of them are human pathogens, with three being plant pathogens (Obase et al., 2016). Several species, including *C. megalosporae*, *C. normandinae* and *C. parmeliae,* were isolated from different lichen species, and were considered lichenicolous fungi (Harutyunyan et al., 2008; Diederich, 2010; Diederich et al., 2013, 2018).

Lichenicolous fungi usually reside within lichens as parasites, and many are broad-spectrum pathogens, saprotrophs, or commensals (Lawrey and Diederich, 2003; Diederich et al., 2018). So far, approximately 2000 obligatory lichenicolous species and over 60 facultative lichenicolous species have been identified (Diederich et al., 2018). These species are categorized into 10 classes, 55 orders, 115 families, and 397 genera (Diederich et al., 2018). Around 50 lichenicolous fungi have been reported from China. It is estimated that the number of lichenicolous fungal species can exceed over 425 in China (Hawksworth and Mariette, 2003; Kondratyuk et al., 2013, 2016a,b, 2017, 2020).

To explore the fungal diversity associated with lichens, we conducted several field surveys in two climatic zones of China, the Inner Mongolia Autonomous Region and Yunnan Province, between 2019 and 2020. During these surveys, we collected various species of lichens from these regions. We isolated an assortment of fungi from the medullary tissues of these lichens. Preliminary morphological and molecular identification of these fungi using the ITS gene region, we identified 15 isolates of black fungi. Further molecular and phylogenetic analyses revealed that these isolates belonged to the genus *Cladophialophora*, but did not represent any of the previously described species. Therefore, we classified these 15 isolates as nine new species within the genus *Cladophialophora*.

## 2. Materials and methods

### 2.1. Collections of lichens and isolations of fungi

A total of 128 lichen samples from nine species (*Flavoparmelia caperata*, *Flavopunctelia flaventior*, *Heterodermia pseudospeciosa*, *Hypotrachyna sinuosa*, *Hypotrachyna vexans*, *Parmelia* sp., *Parmotrema reticulatu*, *Punctelia borreri*, and *Xanthoparmelia tinatina*) were collected from Inner Mongolia Autonomous Region (44°14′2.86″ N, 118°41′35.92″ E) and Yunnan Province (25°43′18.44” N, 101°19′27.84″ E) of China from 2019 to 2020. Each lichen thallus was individually stored in paper bags at 4°C inside a cooler box and brought back to the laboratory for fungal isolations.

All lichen thalli were repeatedly rinsed with sterile denoised water and surface sterilized for 10 min under UV lamp. Using a Leica Zoom 2000 stereo microscope, the upper cortex of the thallus was scraped off and pieces of medullary tissues were rinsed in sterile deionized water. These tissue pieces were placed on potato dextrose agar (PDA, Qingdao Hope Bio-Technology Co., Ltd., China) amended with 0.05% streptomycin (Sangon Biotech (Shanghai) Co., Ltd., China).

All Petri plates were incubated at 25°C for 10 days in darkness. Mycelia emerging from medullary tissue pieces were sub-cultured onto fresh PDA plates. After that, pure cultures were obtained using single hyphal tip technique. All the ex-holotype cultures of new fungal species described in this study were deposited in the China General Microbiological Culture Collection Center (CGMCC), Beijing, China. The holotype specimens were deposited in the Institute of Microbiology (HMAS), Beijing, China (Accession numbers are listed in Table 1). The lichen thalli were deposited in the Collection of Shandong Normal University (SD).

**Table 1:** List of *Cladophialophora* species used for phylogenetic study.

Taxon	Strains	Origin	Source	GenBank accession numbers				
				ITS	LSU	SSU	TUB	TEF
*C. abundans*	CBS126736^**T**^	Brazil	Thorn of Japecanga plant	KC776592	KC812100	–	–	–
*C. aquatica*	MFLUCC 21-0108^**T**^	Thailand	Submerged decaying wood in a freshwater habitat	MT864355	MT860433	–	–	–
*C. arxii*	CBS306.94^**T**^	Germany	Human	EU103986	KX822320	AJ232948	–	EU140593
*C. australiensis*	CBS112793^**T**^	Australia	Apple juice	EU035402	–	KX822275	–	–
*C. bantiana*	CBS173.52^**T**^	USA	Man	EU103989	KF155189	AY554284	–	EU140585
*C. behniae*	CBS146975^**T**^	South Africa	Leaves of *Behnia* sp.	MZ064414	MZ064471	–	MZ078259	MZ078222
*C. boppii*	CBS126.86^**T**^	Brazil	Skin lesion on limb, Man	EU103997	FJ358233	FJ358301	–	EU140596
*C. bromeliacearum*	URM 8085^**T**^	Brazil	Leaves of *Tillandsia catimbauensis*	MW794272	MW794274	–	MW810487	–
*C. cabanerensis*	CBS146718^**T**^	Spain	Soil a wet heathland	NR169978	NG073760	–	–	–
*C. carrionii*	CBS160.54 ^**T**^	Australia	Human	EU137266	FJ358234	FJ358302	EU137201	EU137210
*C. chaetospira*	CBS491.70	Denmark	*Picea abies*	EU035405	EU035405	KX822276	–	–
	CBS514.63			MH858340	MH869959	–	KF928577	–
*C. devriesii* ^T^	CBS147.84^**T**^	USA	Human	EU103985	KC809989	KF155192	–	EU140595
*C. emmonsii*	CBS979.96^**T**^	USA	Human	EU103996	–	–	–	EU140583
*C. eucalypti*	CBS145551^**T**^	Australia	*Eucalyptus dunnii*	NR165555	NG067877	–	–	–
*C. exuberans*	CMRP1227^**T**^	Brazil	From decaying shell of *Orbignya phalerata*	KY680429	KY570931	–	KY689826	–
*C. flavoparmeliae* sp. nov.	CGMCC3.20361^**T**^	China	*Flavoparmelia caperata*	OP828646	OP828622	OP828606	OP857261	OP831203
*C. floridana*	ATCC TSD-8^**T**^	USA	Sclerotium of *Cenococcum geophilum*	AB986343	AB986343	–	–	LC057384
*C. guttulata* sp. nov.	CGMCC3.24219^**T**^	China	*Hypotrachyna sinuosa*	OP828644	OP828620	OP828604	OP857259	OP831198
*C. heterodermiae* sp. nov	CGMCC3.20428^**T**^	China	*Heterodermia pseudospeciosa*	OP828650	OP828625	OP828609	OP857264	–
*C. heterodermiae* sp. nov	CX19Da	China	*Heterodermia pseudospeciosa*	OP828649	–	–	–	–
*C. holosericea* sp. nov.	CGMCC3.20360^**T**^	China	*Hypotrachyna vexans*	OP828643	OP828619	OP828603	OP857258	OP831196
*C. holosericea* sp. nov.	CX08B15	China	*Hypotrachyna vexans*	OP828645	OP828621	OP828605	OP857260	OP831197
*C. hostae*	CBS121637^**T**^	Korea	*Hosta plantaginea*	KX822478	–	KX822277	–	–
*C. humicola*	CBS117536^**T**^	Germany	Soil	EU035408	KC809987	KJ636038	–	–
*C. immunda*	CBS834.96^**T**^	USA	Human	EU137318	KC809990	KF155194	EU137203	EU137257
*C. inabaensis*	EUCL1^**T**^	Japan	Soil	LC128795	LC128795	–	–	–
*C. lanosa*	KNU16:032^**T**^	Korea	Soil	LC387460	LC387461	–	–	–
*C. lichenis* sp. nov	CGMCC3.20424^**T**^	China	*Xanthoparmelia tinatina*	OP828652	OP828627	OP828611	OP857266	OP831194
*C. lichenis* sp. nov.	CGMCC3.20362	China	*Heterodermia pseudospeciosa*	OP828651	OP828626	OP828610	OP857265	OP831193
*C. matsushimae*	MFC-1P384^**T**^	Peru	Palm tree	FN549916	FN400758	–	–	–
*C. minourae*	CBS556.83 ^**T**^	Japan	Decaying wood	AY251087	FJ358235	FJ358303	–	EU140598
*C. minutissima*	UAMH 10709^**T**^	Canada	*Polytrichum juniperinum*	NR137550	–	EF016370	–	–
*C. modesta*	CBS985.96^**T**^	USA	Human	KF928421	KF928485	AJ232953	KF928549	–
*C. moniliformis* sp. nov.	CGMCC3.20346^**T**^	China	*Heterodermia pseudospeciosa*	OP828642	OP828618	OP828602	OP857257	OP831192
*C. mongoliae* sp. nov.	CGMCC3.20305^**T**^	China	*Parmelia* sp.	OP828648	OP828624	OP828608	OP857263	OP831195
*C. multiseptata*	CBS136675^**T**^	Spain	Soil	HG003668	HG003671	–	–	–
*C. mycetomatis*	CBS122637^**T**^	Mexico	Human	FJ385276	KX822321	KX822278	–	–
*C. nyingchiensis*	CGMCC3.17330^**T**^	China	Rock	MG012699	MG197824	MG012728	MG012747	MG012706
	CGMCC3.17514	China	Rock	MG012701	MG197826	MG012730	MG012749	MG012708
	CGMCC3.17329	China	Rock	MG012700	MG197825	MG012729	MG012748	MG012707
*C. olivacea* sp. nov.	CGMCC3.20359^**T**^	China	*Heterodermia pseudospeciosa*	OP828647	OP828623	OP828607	OP857262	OP831199
*C. parmeliae*	CBS129337	Spain	*Hypotrachyna imbricatula*	JQ342180	JQ342182	–	–	–
*C. potulentorum*	CBS115144^**T**^	Australia	Apple juice	DQ008141	–	–	–	–
*C. proteae*	CBS111667^**T**^	South Africa	*Protea cynaroides*	EU035411	EU035411	KJ636039	–	–
*C. psammophila*	CBS110553^**T**^	Netherlands	Soil polluted with petroleum	AY857517	NG058955	AY150798	–	–
*C. pseudocarrionii*	CBS138591^**T**^	Spain	Soil	KU705827	KU705844	–	–	
*C. pucciniophila*	KACC 43957^**T**^	Korea	*Persicaria fauriei*	NR137769	JF263534	–	–	–
*C. recurvata*	FMR16667^**T**^	Spain	Wine	NR172270	LT985879	–	–	–
*C. samoensis*	CBS259.83^**T**^	USA, Samoa	Human	EU137291	KC809992	KX822281	EU137174	EU137233
*C. saturnica*	CBS118724^**T**^	Brazil	Human	EU103984	–	–	–	EU140602
*C. scillae*	CBS116461	New Zealand	*Scilla peruviana*	EU035412	–	KJ636040	–	–
*C. subtilis*	CBS122642^**T**^	Netherlands	Tea drink	FJ385273	KX822322	KX822283	–	–
*C. sylvestris*	CBS350.83^**T**^	Netherlands	*Pinus sylvestris*	EU035413	–	KJ636041	–	–
*C. tengchongensis*	CGMCC3.15201^**T**^	China	Rock	MG012702	MG197827	MG012731	MG012750	MG012709
*C. tortuosa*	ATCC TSD-9^**T**^	USA	*Ceno geophilum*	AB986424	AB986424	–	–	LC057386
*C. tumbae*	JCM28749^**T**^	Japan	Plastic surface	LC192107	LC192072	–	–	–
*C. tumulicola*	JCM 28766^**T**^	Japan	Ceiling stone	LC192098	LC192063	–	–	–
*C. yegresii*	CBS114405^**T**^	Venezuela	*Stenocereus griseus*	EU137322	KX822323	KX822284	EU137209	EU137262
*C. yunnanensis* sp. nov.	CGMCC3.20425^**T**^	China	*Punctelia borreri*	OP828638	OP828615	OP828600	OP857254	OP831200
*C. yunnanensis* sp. nov.	CX37A11	China	*Flavopunctelia flaventior*	OP828639	–	–	–	–
*C. yunnanensis* sp. nov.	CX39A4	China	*Parmotrema reticulatum*	OP828640	OP828616	OP828601	OP857255	OP831201
*C. yunnanensis* sp. nov.	CX39D4	China	*Parmotrema reticulatum*	OP828641	OP828617	–	OP857256	OP831202
*Cyphellophora reptans*	CBS_113.85^**T**^			NR121346	NG067426	NG062867	KC455233	–

*Cyphellophora reptans* served as the outgroup.

Taxon name with a suffixed with *T* indicates ex-type culture.

### 2.2. DNA extraction, PCR amplification, and sequencing

Genomic DNA from all fungal isolates were extracted using PrepMan Ultra Sample Preparation Reagent (Applied Biosystems, California, United States) or CTAB protocol (Zhang et al., 2010). The complete internal transcribed spacer (ITS), partial large subunit of nuclear ribosomal RNA gene (LSU), small subunit of nuclear ribosomal RNA gene (SSU), and translation elongation factor (TEF), and partial β-tubulin gene (TUB) were amplified using primers ITS1/ITS4 (White et al., 1990), LROR/LR5 (Vilgalys and Hester, 1990; White et al., 1990), NS1/NS4 (White et al., 1990; Zoller et al., 1999), EF-728F/EF-986R (Carbone and Kohn, 1999) or EF1-2218R (Groenewald et al., 2013), and T1/T2 (O'donnell and Cigelnik, 1997), respectively. Protein-coding genes were only amplified for fungal isolates representing potentially new species identified during the preliminary identification.

Each 25 μL PCR reaction included 1.0 μL DNA template, 1.0 μL each of forward and reverse primers, 12.5 μL of 2 × Taq polymerase mixture which included buffer, dNTPs, and DNA polymerase (Vazyme Biotech Co., Ltd., China), and 9.5 μL of PCR grade H_2_O. The thermal cycler protocol was denaturation at 95°C for 5 min, followed by 30 cycles of 95°C for 30 s, annealing temperatures of 54°C (ITS), 54°C (LSU), 56°C (SSU), 56°C (TEF), 56°C (TUB) for 60 s, and 72°C for 90 s; final extension at 72°C for 10 min. All PCR amplification was conducted using a Bio-Rad T100™ Thermal Cycler. PCR products were visualized using agarose gel electrophoresis.

All PCR products were sequenced by the Sangon Bioengineering (Shanghai) Co., Ltd. The resulting sequences were assembled using Geneious v.10.2.2 (Biomatters, Auckland, New Zealand). Preliminary identification of the isolates was done using BLAST (Altschul et al., 1990). All sequences of novel fungal species obtained in this study were deposited in the NCBI Gene Bank (Table 1).

### 2.3. Phylogenetic analyses

For phylogenetic analyses, ex-type sequences of ITS, LSU, SSU, TUB, and TEF for all *Cladophialophora* species were retrieved from the NCBI GenBank if available. The final datasets included sequences generated in this study and those retrieved from GenBank. All the datasets were individually aligned using MAFFT v. 7 (Katoh and Standley, 2013) and manually adjusted using MEGA v. 10.2.0 (Kumar et al., 2018). Individual gene regions were phylogenetically analyzed using the method described below, followed by the compilation and analysis of a concatenated dataset.

Phylogenetic analyses of single gene and concatenated datasets were done using three approaches. These were maximum likelihood (ML), maximum parsimony (MP), and Bayesian inference (BI). Software for ML and BI analyses were accessed through the CIPRES Science Gateway v. 3.3^1^ (Miller et al., 2011). The best evolution models of each dataset were estimated using jModelTest2 (Darriba et al., 2012). ML analyses were conducted using RAxML-HPC2 with GTR + GAMMA as the substitution model and 1,000 bootstrap replications (Stamatakis, 2014). MrBayes v. 3.2.7 (Ronquist et al., 2012) was used for the BI analyses with the best substitution model TIM1 + I + G for SSU, TIM2ef + I + G for ITS, TrN + I + G for LSU, TPM2uf + I + G for TUB, TrNef+G for TEF, and SYM + I + G for concatenated data set. Four MCMC chains were run from a random starting tree. Twenty million generations were run with trees sampled at every 100 generations, resulting in 200,000 trees. Tracer v. 1.7^2^ was used to determine the chain convergence and the effective sample size values. We discarded a quarter of sampled trees (50,000) during burn-in. Posterior probabilities were calculated from the remaining trees. MEGA v. 10.2.0 (Kumar et al., 2018) was used to for the MP analyses with 1,000 bootstrap replicates, gaps were treated as a fifth state character. FigTree v. 1.4.3 was used to visualize and edit trees. All the datasets and trees were submitted to the TreeBase (Study ID 29976).

### 2.4. Morphology and culture characteristics

For morphological studies, all isolates of the new fungal species identified in this study were used. Colony morphologies of potentially new fungal species were described from 21-day-old cultures growing at 25°C on PDA. Micro-morphological structures of the isolates were visualized and photographed using a Leica DFC95 camera attached to a Leica DM6 microscope. ImageJ v. 1.53u^3^ was used for measuring taxonomically relevant features. At least 40 measurements were recorded for each morphological feature, and the statistics were presented as (minimum –) mean – standard deviation – mean + standard deviation (−maximum).

Twenty-one-day-old fungal isolates grown on PDA at 25°C were utilized for the growth study. Agar plugs measuring 5 mm in diameter were obtained from the culture and placed in 90 mm Petri dishes containing PDA. Three replicate plates were prepared for each fungal isolate, spanning temperatures from 5 to 35°C at 5°C intervals (± 0.5°C). Following a 28-day period, the colony diameters of each isolate were measured.

## 3. Results

### 3.1. Collections of lichens and isolations of fungi

A preliminary identification of 1,587 fungal isolates using ITS, LSU, and SSU sequences revealed that 15 isolates recovered from lichens were *Cladophialophora* species. Among these, five fungal isolates were recovered from Inner Mongolia Autonomous Region whereas ten from the Yunnan Province (Table 1). For a majority of the isolates, the ITS sequence had a similarity lower than 98% with all previously described *Cladophialophora* species. This suggested that among those isolates were some potentially unknown species.

### 3.2. Phylogenetic analyses

**Figure 1:**
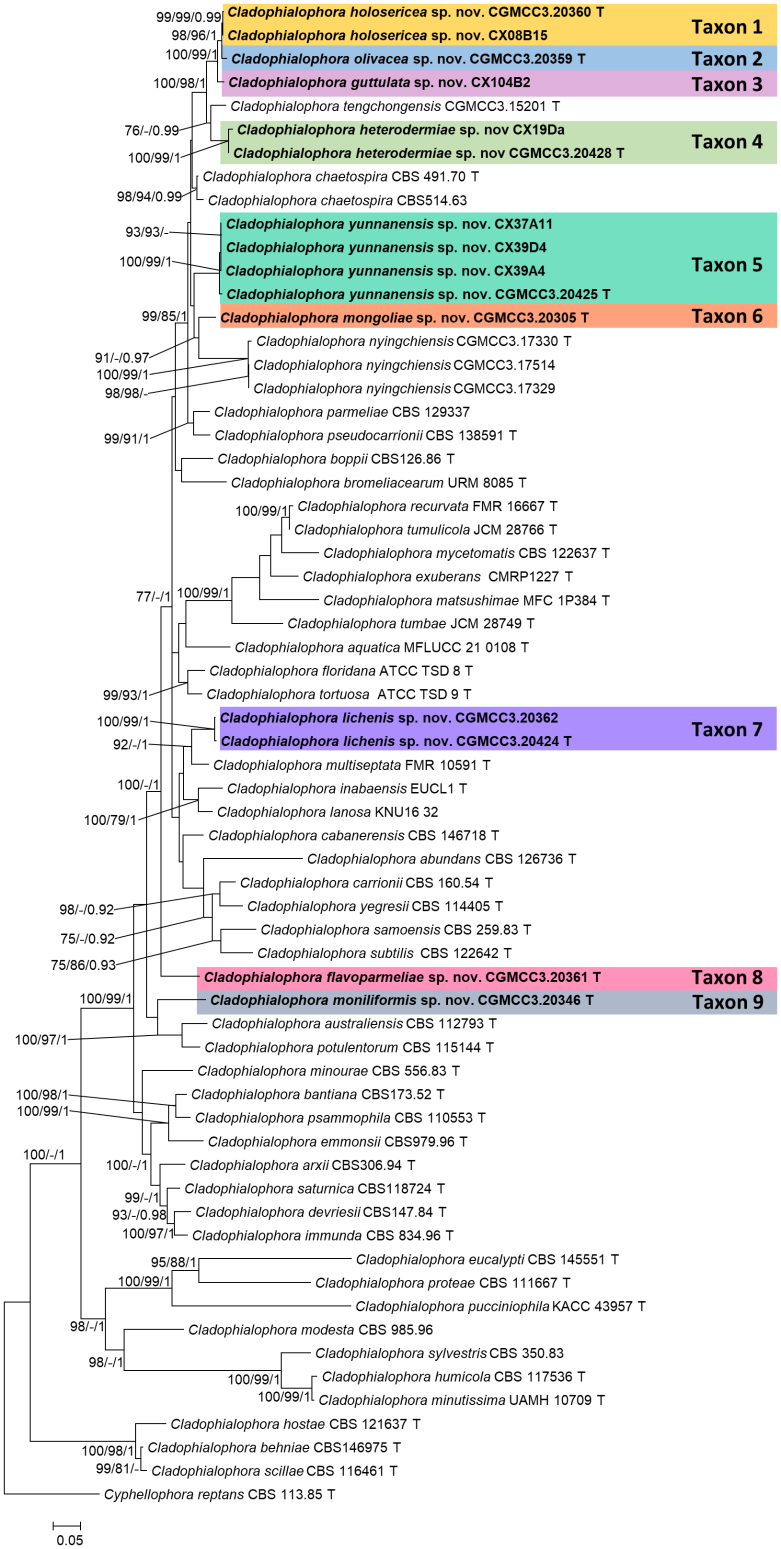
Maximum likelihood tree of *Cladophialophora* species constructed using the concatenated dataset (ITS+LSU + SSU + TUB+TEF). Bootstrap support values ≥75% and posterior probabilities ≥0.90 are indicated above the nodes as ML/MP/PP, respectively. Isolates obtained in this study are in bold font. T = ex-type isolates.

The topologies of the trees derived from the phylogenetic analyses of single gene and concatenated datasets were not identical due to unequal taxon sampling (Figure 1, Supplementary Figures S1−S5). Henceforth, we used the ML tree emerging from the analyses of concatenated dataset for delineation of *Cladophialophora* species recovered in this study (Figure 1). This concatenated data included 63 taxa (Table 1) and 3,199 characters, SSU 1–913, ITS 914–1,651, LSU 1652–2,487, TUB 2488–2,955, and TEF 2956–3,199. Bootstrap values ≥75% and posterior probability ≥0.90 were considered reliable (Figure 1, Supplementary Figures S1−S5).

**Figure 2:**
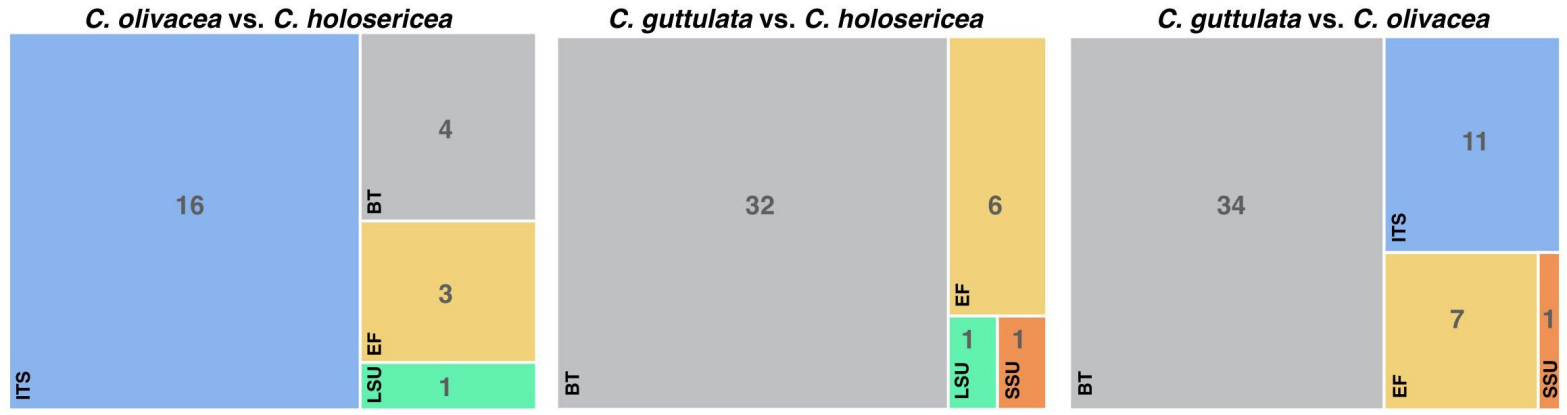
The sequence variability between *Cladophialophora holosericea*, *Cladophialophora olivacea* and *Cladophialophora guttulata*.

In the concatenated tree, *Cladophialophora* isolates CGMCC3.20360, CX08B15, CGMCC3.20359, and CGMCC3.24219 formed a monophyletic lineage that emerged as the sister to a clade that included *Cladophialophora tengchongensis* and Taxon 4 (see below). Among these four isolates, the monophyly of CGMCC3.20360 and CX08B15 (Taxon 1) received significant statistical support (ML/MP/BI: 97/99/0.99; Figure 1). Isolate CGMCC3.20359 (Taxon 2) emerged as the sister taxon to Taxon 1 (Figure 1). CGMCC3.24219 (Taxon 3) was sister to the clade that included Taxon 1 and Taxon 2 (Figure 1). Even though the monophyly of these four isolates obtained considerable branch support in the concatenated tree, there was still a significant difference in the gene regions between the taxa (Figure 2).

Isolates CX19Da and CGMCC3.20428 (Taxon 4) emerged as the sister taxa to *C. tengchongensis* in concatenated tree (Figure 1). However, this relationship varied between the single gene trees (Supplementary Figures S1−S5). We were unable to amplify the TEF of the isolate CGMCC3.20428, as well as the SSU, LSU, TUB, and TEF of CX19Da. In ITS sequences, there were three base pairs differences between isolates of Taxon 4.

In the concatenated tree, four isolates of Taxon 5 (CGMCC3.20425, CX37A11, CX39A4, and CX39D4) emerged as a monophyletic lineage with significant branch support (ML/MP/BI: 100/100/1) and sister to a clade that included Taxon 1, 2, 3, 4, and *C. tengchongensis* (Figure 1). However, the latter relationship did not receive significant branch support. Hence, it varied between the single gene trees (Supplementary Figures S1, S2, S4). Additionally, between the isolates of Taxon 5, we detected some polymorphisms. There were a 16-bp differences in TUB between the pairs CX39A4/CGMCC3.20425 and CX39D4/CGMCC3.20425.

In the concatenated, ITS, and TEF trees, isolate CGMCC3.20305 (Taxon 6) emerged as sister to *C. nyingchiensis* (Figure 1, Supplementary Figures S1, S5). This relationship was statistically significant (ML/MP/BI: 91/−/0.97).

In the concatenated tree, isolates CGMCC3.20424 and CGMCC3.20362 (Taxon 7) formed a monophyletic clade with high statistical support values (ML/MP/BI: 100/100/1) and emerged as the sister to *C. multiseptata* with moderate statistical support (ML/MP/BI: 75/−/1; Figure 1). However, this relationship was inconsistent across the single gene trees (Supplementary Figures S1−S5). We also identified some polymorphism between the isolates CGMCC3.20424 and CGMCC3.20362 (ITS 6, SSU 1, TEF 3, TUB 1).

In the concatenated tree, CGMCC3.20361 (Taxon 8) did not group with any known species of *Cladophialophora* (Figure 1). This relationship was echoed in the LSU, SSU, and TEF trees (Supplementary Figures S2, S3, S5), but in the ITS and TUB trees, Taxon 8 formed a monophyletic clade with *C. bromeliacearum* (Supplementary Figures S1, S4). None of these relationships received significant branch support.

CGMCC3.20346 (Taxon 9) emerged as a sister taxon to *C. potulentorum* and *C. australiensis* in concatenated tree (Figure 1). However, this relationship was inconsistent across the single gene trees (Supplementary Figures S1−S5).

### 3.3. Taxonomy

Taxon 1

***Cladophialophora holosericea*** H.L. Si, R.L. Chang, T. Bose and X.X. Zheng, sp. nov.

Figures 3A−D.

**Figure 3:**
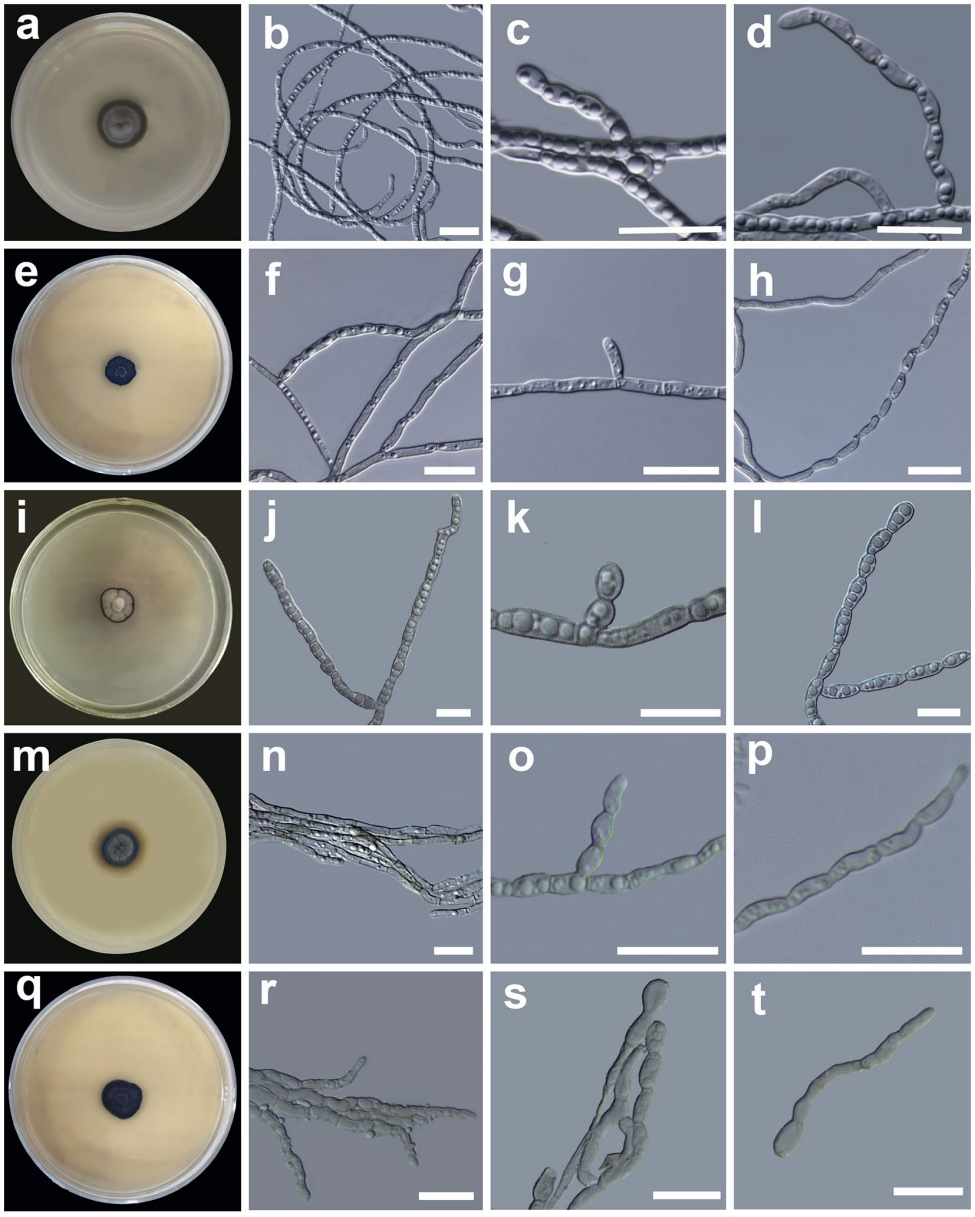
Morphology of *Cladophialophora holosericea* sp. nov. (HMAS 350277) **(A)** 21-day-old culture on a PDA; **(B)** straight and curved hyphae; **(C,D)** lateral conidial chains emerging from hyphae; *Cladophialophora olivacea* sp. nov. (HMAS 350273) **(E)** 21-day-old culture on a PDA; **(F)** bi-guttulate hyphae; **(G)** lateral conidial initial on hyphae; **(H)** chain of terminal conidia; *Cladophialophora guttulata* sp. nov. (HMAS 352295) **(I)** 21-day-old culture on a PDA; **(J)** branched multi-guttulate hyphae; **(K)** lateral conidia; **(L)** conidial chains; *Cladophialophora heterodermiae* sp. nov. (HMAS 350278) **(M)** 21-day-old culture on a PDA; **(N)** hyphae; **(O,P)** conidial chain; *Cladophialophora yunnanensis* sp. nov. (HMAS 350288) **(Q)** 21-day-old culture on a PDA; **(R)** hyphae; **(S)** conidial chains; **(T)** germinating conidia. Scale bars = 10 μm.

MycoBank no.: MB 846410.

*Etymology*: The name refers to the velutinous colony morphology of this fungus on PDA medium.

*Diagnosis*: Circinate hyphae and the smallest conidial dimension distinguish *C. holosericea* from the closely related species.

*Type*: **China**: *Yunnan Province*, Chuxiong Yi Autonomous Prefecture, Chuxiong city, Dayao county (26°32′71.54” N, 100°57′3.6″ E), isolated from the medullary tissue of the lichen *Hypotrachyna vexans* (SDCX08), 13 Nov. 2020, *H. L. Si*, CX08B1 = CGMCC3.20360 (The ex-holotype culture), dried culture HMAS 350277 (holotype specimen), GenBank Accession Numbers: ITS OP828643; LSU OP828619; SSU OP828603; TUB OP857258; and TEF OP831196.

*Description*: *Hyphae* gray, smooth, straight, circinate at the tip, branched, septate, constricted at the septa, compartments cylindrical, usually 2–4 guttulate, measuring 1.1–3.1 μm in diam (Figure 3B). *Conidiogenous cell* compartments cylindrical, usually 2–4 guttulate, measuring (2.9 −)5.3–8.6(− 9.4) × (1.1 −)1.5–2.2(− 3.1) μm. *Conidia* hyaline, surface smooth, lateral in position, usually oval to oblong in shape, sometimes spherical, in short chains of 2–9, measuring (2.3 −)3.0–5.1(− 6.5) × (1.3 −)1.5–3.3(− 3.5) μm (Figures 3C,D). *Chlamydospores* absent. Yeast-like cells absent. Sexual morph unknown.

*Culture characteristics*: The colony morphology on the PDA after 21 days was compact, dark gray in the center with a dark olivaceous gray margin, surface velutinous, convex, and margin entire (Figure 3A). The colony grows slowly on PDA medium, reaching 17 mm in diameter after 4 weeks at 25°C. The optimal growth temperature is 25°C. No growth was observed at 5, 30, and 35°C.

*Habitat*: Each isolate of this fungus was recovered from two separate thalli of the lichen *H. vexans* collected in Yunnan Province of China.

*Note*: *Cladophialophora holosericea* is phylogenetically close to *C. olivacea* and *C. guttulata*. However, these three species have substantial differences in their colony, hyphal and conidial morphologies (Table 2). For example, compared to *C. olivacea* and *C. guttulata*, *C. holosericea* has circinate hyphae and smallest conidial dimension (Table 2). Additionally, there is a significant variation in the SSU, ITS, LSU, TUB, and TEF gene sequences between these three species. There was a total of 24 bps differences between *C. holosericea* and *C. olivacea*, 40 bps between *C. holosericea* and *C. guttulata*, and 53 bps between *C. olivacea* and *C. guttulata* (Figure 2).

**Table 2:** Comparison of biological characteristics between five species of *Cladophialophora* recovered from the present study and *Cladophialophora tengchongensis.*

	*C. holosericea*	*C. olivacea*	*C. guttulata*	*C. heterodermiae*	*C. tengchongensis*	*C. yunnanensis*
	In this study	In this study	In this study	In this study	Sun et al., (2020)	In this study
No. of isolates	2	1	1	2		4
Isolated from	*Hypotrachyna vexans*	*Heterodermia pseudospeciosa*	*Hypotrachyna sinuosa*	*Heterodermia pseudospeciosa*	Rock	*Flavopunctelia flaventior Punctelia borreri, Parmotrema reticulatum*
Colony morphology	Dark gray in color in the centre with a dark olivaceous gray margin, convex, margin entire	Dark olive green, convex, thin dark entire margin, dark striation extending from the colony margin toward the centre	Off-white with distinct blackish brown margin, striation extending from the entire colony margin	Smoky gray with distinct dark-gray wide margin, with striation, margin entire, light brown diffusible pigment around the colony	Pale gray to deep-olivaceous gray, zonate, glossy near the periphery, margin entire	Dark gray in color, slightly convex, margin entire
Colony texture	Velutinous	Villose	Villose	Villose	Smooth	Smooth
Growth rate after four weeks (mm)	17	15	11	9	28	10
Optimal growth temperature (°C)	25	25	25	25	20–25	25
Hyphal Morphology	Gray, smooth, straight or curved, branched	Hyaline, smooth, straight, branched	Light brown, smooth, straight, branched, compartment minutely doliform	Hyaline or brown, smooth, straight, branched, compartment cylindrical	Hyaline to pale olive green, branched, compartment cylindrical to elongate	Light brown, septate, smooth, branched, compartment cylindrical
Guttules per compartment	2–4	2	3–9 or higher	2–4	1–2	2–4
Septal morphology	Constricted	Constricted	Constricted	Constricted	Constricted	Constricted
Septal diam. (μm)	1.1–3.1	1.0–4.5	1.6–3.7	0.8–2.2	1.9–4.0	1.0–2.3
Conidiogenous cell (μm)	2.9–9.4 × 1.1–3.1	2.8–10.5 × 1.0–2.1	2.3–13.6 × 1.6–2.8	3.4–7.4 × 1.2–1.9	–	5.0–12.7 × 1.6–2.8
Conidia position	Lateral	Apical and lateral	Apical and lateral	Apical and lateral	Lateral or terminal	Apical or lateral
Conidia shape	Oval to oblong often spherical	Oblong	Oblong	oval to fusiform	ellipsoidal, fusiform or subcylindrical	Ellipsoid to round
Conidia morphology	In short chain of 2–9 cells	One-celled, moniliform	One-celled, moniliform	Once-celled, often in short branched chain	Once-celled, forming long branched chain	One-celled, often in short chain
Conidium color	Hyaline	Hyaline	Hyaline	Hyaline, brown on maturation	Hyaline to pale olive green	Hyaline
Conidium measurement	3.0–5.1 × 1.5–3.3	3.9–6.4 × 1.2–2.0	5.5–8.9 × 2.4–2.9	3.0–5.7 × 1.2–3.5	4.2–10.6 × 3.1–5.5	3.4–6.6 × 1.1–3.5
Chlamydospore	Absent	Absent	Absent	Absent	Absent	Absent
Sexual morph	Unknown	Unknown	Unknown	Unknown	Unknown	Unknown

*Additional specimen examined*: **China**: *Yunnan Province*, Chuxiong Yi Autonomous Prefecture, Chuxiong city, Dayao county (26°32′71.54” N, 100°57′3.6″ E), isolated from the medullary tissue of the lichen *Hypotrachyna vexans* (SDCX08), 13 Nov. 2020, *H. L. Si*, CX08B15.

Taxon 2

***Cladophialophora olivacea*** H.L. Si, R.L. Chang, T. Bose and X.X. Zheng, sp. nov.

Figures 3E–H.

MycoBank no.: MB 846411.

*Etymology*: The name refers to the olive-green colony color of this fungus on PDA medium.

*Diagnosis*: *Cladophialophora olivacea* can be distinguished from other closely related species by its colony color and conidial dimensions.

*Type*: **China**: *Yunnan Province*, Chuxiong Yi Autonomous Prefecture, Chuxiong city, Dayao county (26°32′71.54” N, 100°57′3.6″ E), isolated from the medullary tissue of the lichen *Heterodermia pseudospeciosa* (SDCX19), 20 Nov. 2020, *H. L. Si*, CX19Dc = CGMCC3.20359 (The ex-holotype culture), dried culture HMAS 350273 (holotype specimen). GenBank Accession Numbers: ITS OP828647; LSU OP828623; SSU OP828607; TUB OP857262; and TEF OP831199.

*Description*: *Hyphae* hyaline, smooth, branched, septate, often constricted at the septa, compartments cylindrical, usually bi-guttulate, measuring 1.0–4.5 μm in diam (Figure 3F). *Conidiogenous cell* compartments cylindrical, usually bi-guttulate, measuring (2.8 −)4.7–9.0(− 10.5) × (1.0 −)1.4–1.8(− 2.1) μm. *Conidia* terminal or lateral in position, hyaline, surface smooth, one-celled, oblong in shape, moniliform, measuring (2.6 −)3.9–6.4(− 8.5) × (0.9 −)1.2–2.0(− 2.1) μm (Figures 3G,H). *Chlamydospores* absent. Yeast-like cells absent. Sexual morph unknown.

*Culture characteristics*: The colony morphology on the PDA after 21 days was compact, dark olive green in color, convex, surface villose, and margin entire (Figure 3E). The colony grows slowly on PDA medium, reaching 15 mm in diameter after four weeks at 25°C. The optimal growth temperature is 25°C. No growth was observed at 5, 30, and 35°C.

*Habitat*: A single isolate of this fungus was recovered from *Heterodermia pseudospeciosa* collected in Yunnan Province of China.

*Note*: *Cladophialophora olivacea* has several morphological and genetic differences with *C. holosericea* and *C. guttulata* (Table 2; Figure 2). This species has a distinct, dark olive-green colony, and the conidial size is larger than that of *C. holosericea* but smaller than that of *C. guttulata* (Table 2).

Taxon 3

***Cladophialophora guttulata*** H.L. Si, T. Bose and R.L Chang, sp. nov.

Figures 3I–L.

MycoBank no.: MB 846412.

*Etymology*: The name refers to the abundance of guttulae in each hyphal compartment.

*Diagnosis*: The abundance of oil bodies (guttules) in the hyphal compartment of *C. guttulata* distinguishes it from other closely related species.

*Type*: **China**: *Yunnan Province*, Chuxiong Yi Autonomous Prefecture, Chuxiong city, Dayao county (26°32′71.54” N, 100°57′3.6″ E), isolated from *Hypotrachyna sinuosa* (SDCX104), 13 Nov. 2020, *H. L. Si*, CX104B2 = CGMCC3.24219 (The ex-holotype culture), dried culture HMAS 352295 (holotype specimen), GenBank Accession Numbers: ITS OP828644; LSU OP828620; SSU OP828604; TUB OP857259; and TEF OP831198.

*Description: Hyphae* light brown, smooth, septate, branched, compartments cylindrical, slightly doliform, 3–9 guttulate, measuring 1.6–3.7 μm in diam, (Figure 3J). *Conidiogenous cell* compartments cylindrical, 3–9 guttulate, measuring (2.3 −)6.5–12.6(− 13.6) × (1.6 −)2.0–2.8(− 2.8) μm. *Conidia* one-celled, or forming short chains of 2–4 cells, hyaline, surface smooth, shape oblong, clavate to oval in shape, usually 3–6 guttulate, measuring (3.3 −)5.5–8.9(− 10.6) × (2.0 −)2.4–2.9(− 3.1) μm (Figures 3K,L). *Chlamydospores* absent. Yeast-like cells absent. Sexual morph unknown.

*Culture characteristics*: The colony morphology on the PDA after 21 days was compact, off-white in color, with thin dark entire margin, convex, surface villose, dark striation extending from the colony margin toward the center (Figure 3I). The colony grows slowly on PDA, reaching 11 mm in diam after 4 weeks at 25°C. The optimal growth temperature is 25°C. No growth was observed at 5, 30, and 35°C.

*Habitat*: The isolates of this fungus was recovered from *Hypotrachyna sinuosa* collected in Yunnan Province of China.

*Note*: *Cladophialophora guttulata* is distinguished from *C. holosericea* and *C. olivacea* by its distinctive colony morphology and abundance of oil bodies (guttules) in hyphal compartments and conidia (Table 2, Figure 2).

Taxon 4

***Cladophialophora heterodermiae*** H.L. Si, R.L. Chang, T. Bose and X.X. Zheng, sp. nov.

Figures 3M–P.

MycoBank no.: MB 846413.

*Etymology*: The name refers to its lichen host *Heterodermia pseudospeciosa* from which a single isolate of this fungus was recovered.

*Diagnosis*: *Cladophialophora heterodermiae* differs from its closely related species, *C. tengchongensis*, in regards to growth rates and colony morphology.

*Type*: **China**: *Yunnan Province*, Chuxiong Yi Autonomous Prefecture, Chuxiong city, Dayao county (26°32′71.54” N, 100°57′3.6″ E), isolated from the medullary tissue of *Heterodermia pseudospeciosa* (SDCX19), 20 Nov. 2020, *H. L. Si*, CX19Dd = CGMCC3.20428 (ex-holotype culture), dried culture HMAS 350278 (holotype specimen). GenBank Accession Numbers: ITS OP828650; LSU OP828625; SSU OP828609; and TUB OP857264.

*Description*: *Hyphae* septate, smooth, brown to hyaline in color, branched, compartments cylindrical, 2–4 guttulate, slightly constricted at the septa, measuring 0.8–2.2 μm in diam (Figure 3N). *Conidiogenous cell* compartments cylindrical, 2–4 guttulate, measuring (3.4 −)3.9–6.4(− 7.4) × (1.2 −)1.4–1.8(− 1.9) μm. *Conidia* are apical or lateral in position, surface smooth, oval to slightly curved in shape, hyaline or brown in color, forming short (2–3 cells) to long (5–8 celled) chain, occasionally branched chains, measuring (2.0 −)3.0–5.7(− 8.1) × (1.1 −)1.2–3.5(− 4.2) μm (Figures 3O,P). *Chlamydospores* absent. Yeast-like cells absent. Sexual morph unknown.

*Culture characteristics*: The colony morphology on the PDA after 21 days was compact, smoky gray in color, with a dark-gray wide entire margin, surface villose, with distinct striation, light brown diffusible pigment around the colony (Figure 3M). Colony grows slowly on PDA, reaching 9 mm in diam after four weeks at 25°C. The optimal growth temperature is 25°C. No growth was observed at 5, 30, and 35°C.

*Habitat*: The isolate of this fungus was recovered from *Heterodermia pseudospeciosa* collected in Yunnan Province of China.

*Note*: *Cladophialophora heterodermiae* is phylogenetically close to *C. tengchongensis*. However, these two species have substantial differences in growth rate and colony morphology, septal diameter, conidial morphology, and dimensions (Sun et al., 2020). These two species also have some differences between the ITS, LSU, and TUB gene regions: 36–40 bps in ITS, 10 bps in LSU, and 63 bps in TUB.

*Additional specimen examined*: **China**: *Yunnan Province*, Chuxiong Yi Autonomous Prefecture, Chuxiong city, Dayao county (26°32′71.54” N, 100°57′3.6″ E), isolated from *Heterodermia pseudospeciosa* (SDCX08), 05 Dec. 2020, H. L. Si, CX19Da.

Taxon 5

***Cladophialophora yunnanensis*** H.L. Si, R.L. Chang, T. Bose and X.X. Zheng, sp. nov.

Figures 3Q–T.

MycoBank no.: MB 846414.

*Etymology*: The name of this fungus refers to Yunnan Province of China.

*Diagnosis*: *Cladophialophora yunnanensis* differs from closely related species in terms of colony morphology, hyphal morphology, and conidial shape and size.

*Type*: **China**: *Yunnan Province*, Chuxiong Yi Autonomous Prefecture, Chuxiong city, Dayao county (26°32′71.54” N, 100°57′3.6″ E), isolated from the medullary tissue of the lichen *Punctelia borreri* (SDCX37), 05 Dec. 2020, *H. L. Si*, CX37A10 = CGMCC3.20425 (The ex-holotype culture), dried culture HMAS 350288 (holotype specimen). GenBank Accession Numbers: ITS OP828638; LSU OP828615; SSU OP828600; TUB OP857254; and TEF OP831200.

*Description*: *Hyphae* branched, septate, light brown, composed by cylindrical to elongate cells, 2–4 guttulate, constricted at the septa, measuring 1.0–2.3 μm in diam (Figure 3R). *Conidiogenous cell* cylindrical, 2–4 guttulate, measuring (5.0 −)5.3–9.1(− 12.7) × (1.6 −)1.8–2.4(− 2.8) μm. *Conidia* one-celled or in short chain of 2–3 cells, lateral or terminal in position, ellipsoid to round in shape, usually 2–3 guttulate, surface smooth, aseptate, measuring (2.4 –)3.4–6.6(− 9.6) × (0.9 −)1.1–3.5(− 4.1) μm (Figures 3S,T). *Chlamydospores* absent. Yeast-like cells absent. Sexual morph unknown.

*Culture characteristics*: The colony morphology on the PDA after 21 days was compact, dark gray in color, margin entire, slightly raised in the center, and surface smooth (Figure 3Q). The colony grows slowly on PDA, reaching 10 mm in diam after four weeks at 25°C. The optimal growth temperature is 25°C. No growth was observed at 5, 30, and 35°C.

*Habitat*: Associated with three species of lichen, *Flavopunctelia flaventior, Punctelia borreri*, and *Parmotrema reticulatum*, in the Yunnan Province of China.

*Note*: *Cladophialophora yunnanensisis* sister to the clade that includes *C. holosericea*, *C. olivacea*, *C. guttulata*, *C. heterodermiae*, and *C. tengchongensis*. The colony morphology, hyphal morphology, and conidial shape and size of this species are distinct from those of its sister species (Table 2).

*Additional specimens examined*: **China**: *Yunnan Province*, Chuxiong Yi Autonomous Prefecture, Chuxiong city, Dayao county (26°32′71.54” N, 100°57′3.6″ E), isolated from *Flavopunctelia flaventior* (SDCX371), 05 Dec. 2020, *H. L. Si,* CX37A11; **China**: *Yunnan Province*, Chuxiong Yi Autonomous Prefecture, Chuxiong city, Dayao county (26°32′71.54” N, 100°57′3.6″ E), isolated from *Parmotrema reticulatum* (SDCX39), 05 Dec. 2020, *H. L. Si*, CX39A4; **China**: *Yunnan Province*, Chuxiong Yi Autonomous Prefecture, Chuxiong city, Dayao county (26°32′71.54” N, 100°57′3.6″ E), isolated from *Parmotrema reticulatum* (SDCX39), 05 Dec. 2020, *H. L. Si*, CX39D4.

Taxon 6

***Cladophialophora mongoliae*** H.L. Si, T. Bose and R.L. Chang, sp. nov.

Figures 4A–D.

**Figure 4:**
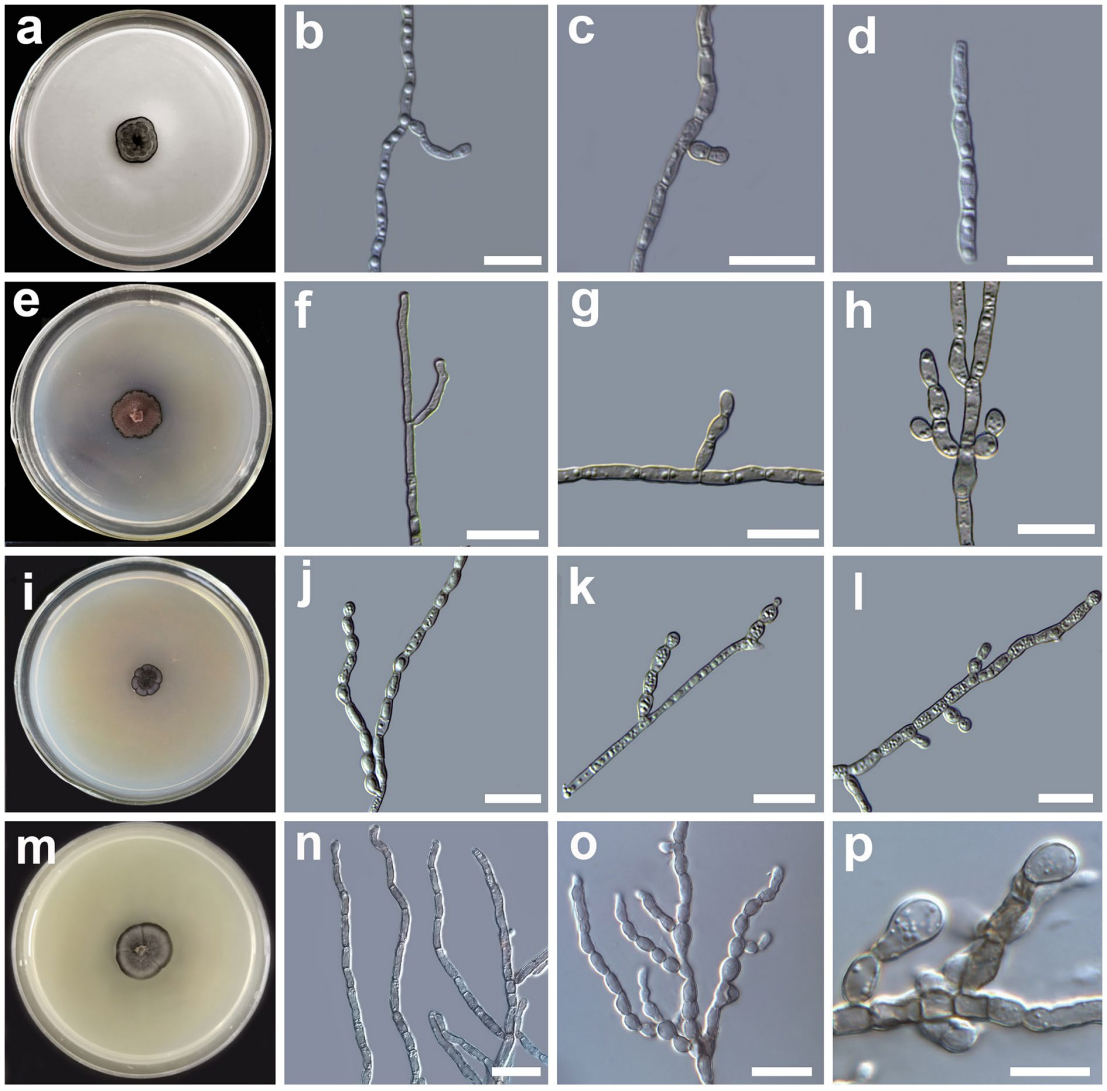
Morphology of *Cladophialophora mongoliae* sp. nov. (HMAS 350287) **(A)** 21-day-old culture on a PDA; **(B)**, branched hyphae, **(C)** lateral conidial initial on hyphae; **(D)** conidial chains; *Cladophialophora lichenis* sp. nov. (HMAS 350280) **(E)** 21-day-old culture on a PDA; **(F)** branched hyphae; **(G)** lateral chain of conidia; **(H)** apical conidial chains; *Cladophialophora flavoparmeliae* sp. nov. (HMAS 350291) **(J)** branched hyphae; **(K,L)** apical and lateral chain of conidia; *Cladophialophora moniliformis* (HMAS 350276) **(M)** 21-day-old culture on a PDA; **(N)** branched hyphae; **(O)** moniliform branched conidia chains; **(P)** lateral chain of conidia emerging from hyphae. Scale bars = 10 μm.

MycoBank no.: MB 846415.

*Etymology*: The name refers to the Inner Mongolia Autonomous Region of China, where the fungus was isolated.

*Diagnosis*: *Cladophialophora mongoliae* differs from *C. nyingchiensis* morphologically by having smaller conidia.

*Type*: **China**: *Inner Mongolia Autonomous Region*, Chifeng city, Balin Right Banner, Mt. Qingyangcheng (44°14′2.86″ N, 118°41′35.92″ E), isolated from the medullary tissue of *Parmelia* sp. (SDNM423), 7 Jul. 2019, *H. L. Si*, 423c = CGMCC3.20305 (The ex-holotype culture), dried culture HMAS 350287 (holotype specimen). GenBank Accession Numbers: ITS OP828648; LSU OP828624; SSU OP828608; TUB OP857263; and TEF OP831195.

*Description*: *Hyphae* brown, smooth, straight or minutely curved, branched, septate, constricted at the septa, compartments cylindrical, usually 2–4 guttulate, measuring 1.1–1.9 μm in diam (Figures 4B,C). *Conidiogenous cell* compartments cylindrical, 2–4 guttulate, measuring (4.8 −)5.3–8.6(− 9.5) × (1.1 −)1.5–1.6(− 1.9) μm. *Conidia* hyaline, surface smooth, lateral in position emerging from undifferentiated hyphae, usually oval to elliptic to cylindrical in shape, usually 2–3 guttulate, one-celled or in short chains of 2–4 cells, measuring (3.9 −)4.8–6.4(− 6.8) × (1.2 −)1.3–1.7(− 2.0) μm (Figures 4C,D). *Chlamydospores* absent. Yeast-like cells absent. Sexual morph unknown.

*Culture characteristics*: The colony morphology on the PDA after 21 days was compact, gray to dark gray in color with distinct black entire margin center convex (Figure 4A). The colony grows slowly on PDA medium, reaching 15 mm in diam after four weeks at 25°C. The optimal growth temperature is 25°C. No growth was observed at 5 and 35°C.

*Habitat*: Medullary tissue of the lichen of *Parmelia* sp. collected from Inner Mongolia Autonomous Region.

*Note*: In our phylogenetic analyses, *C. mongoliae* emerged as the sister species to *C. nyingchiensis* (Figure 1). This relationship was supported by a significant statistical value. Simultaneously, *C. mongoliae* also has a substantial morphological difference with *C. nyingchiensis*. For example, the average dimension of the conidium, *C. mongoliae* (4.8–6.4 × 1.3–1.7 μm), *C. nyingchiensis* (6.5–22.1 × 1.3–3.2 μm) (Sun et al., 2020).

Taxon 7

***Cladophialophora lichenis*** H.L. Si, R.L. Chang, T. Bose and X.X. Zheng, sp. nov.

Figures 4E–H.

MycoBank no.: MB 846416.

*Etymology*: The name refers to lichen, from which this fungus was isolated.

*Diagnosis*: *Cladophialophora lichenis* can be distinguished from its closely related species, *C. multiseptata*, by the presence of aseptate conidia.

*Type*: **China**: *Inner Mongolia Autonomous Region*, Chifeng city, Balin Right Banner, Mt. Qingyangcheng (44°14′2.86″ N, 118°41′35.92″ E), isolated from the medullary tissue of *Xanthoparmelia tinatina* (SDNM420), 7 Jul. 2019, *H. L. Si*, 420 = CGMCC3.20424 (The ex-holotype culture), dried culture HMAS 350280 (holotype specimen). GenBank Accession Numbers: ITS OP828652; LSU OP828627; SSU OP828611; TUB OP857266; and TEF OP831194.

*Description*: *Hyphae* hyaline to brown in color, smooth, straight or minutely curved, branched, septate, constricted at the septa, compartments cylindrical, usually 2–6 guttulate (if more usually guttules smaller in size), measuring 0.9–2.7 μm in diam (Figure 4F). *Conidiogenous cell* compartments cylindrical, 2–6 guttulate, measuring (6.5 −)6.8–11.0(− 13.2) × (1.5 −)1.8–2.5(− 2.7) μm. *Conidia* brown in color, surface smooth, lateral in position emerging from undifferentiated hyphae, usually oval to spherical in shape, guttulate, one-celled or in short chains of 2–4 cells, branched, measuring (1.4 −)2.1–6.7(− 9.4) × (2.3 −)2.6–4.4(− 6.2) μm (Figures 4G,H). *Chlamydospores* absent. Yeast-like cells absent. Sexual morph unknown.

*Culture characteristics*: The colony morphology on the PDA after 21 days was compact, dark pinkish brown in color with a prominent brown irregular margin, surface villose with distinct protuberances in the center (Figure 4E). The colony grows slowly on PDA medium, reaching 16 mm in diam after four weeks at 25°C. The optimal growth temperature is 25°C. No growth was observed at 5 and 35°C.

*Habitat*: Medullary tissue of the lichens *Xanthoparmelia tinatina* and *Heterodermia pseudospeciosa* collected from Inner Mongolia Autonomous Region of China.

*Note*: In our phylogenetic analyses, *C. lichenis* emerged as the sister species to *C. multiseptata* (Figure 1). However, this relationship did not receive significant statistical support. Besides this, *C. lichenis* and *C. multiseptata* have substantial morphological differences. The conidia of *C. lichenis* are aseptate, whereas those of *C. multiseptata* are asepate or uniseptate (Crous et al., 2013). The average conidial size of *C. lichenis* (2.1–6.7 × 2.6–4.4 μm) is smaller than that of *C. multiseptata* (4.5–18 × 3–5 μm) (Crous et al., 2013).

*Additional specimen examined*: **China**: *Inner Mongolia Autonomous Region*, Chifeng city, Balin Right Banner, Mt. Qingyangcheng (44°14′2.86″ N, 118°41′35.92″ E), isolated from *Heterodermia pseudospeciosa* (SDNM418), 7 Jul. 2019, *H. L. Si*, 418Z = CGMCC3.20362 (The ex-paratype culture), dried culture HMAS 350274 (paratype specimen).

Taxon 8

***Cladophialophora flavoparmeliae*** H.L. Si, R.L. Chang, T. Bose and X.X. Zheng, sp. nov.

Figures 4I–L.

MycoBank no.: MB 846417.

*Etymology*: The name refers to the host lichen species, *Flavoparmelia caperata*, from which a single isolate of this fungus was isolated.

*Diagnosis*: *Cladophialophora flavoparmeliae* has a slow growth rate on PDA, and the colony is dark gray in color.

*Type*: **China**: *Inner Mongolia Autonomous Region*, Chifeng city, Balin Right Banner, Mt. Qingyangcheng (44°14′2.86″ N, 118°41′35.92″ E), isolated from medullary tissue of *Flavoparmelia caperata* (SDNM51), 7 Jul. 2019, *H. L. Si,* y0051 = CGMCC3.20361 (The ex-holotype culture), dried culture HMAS 350291 (holotype specimen). GenBank Accession Numbers: ITS OP828646; LSU OP828622; SSU OP828606; TUB OP857261; and TEF OP831203.

*Description*: *Hyphae* light gray, smooth, straight or slightly curved, branched, septate, constricted at the septa, compartments cylindrical, usually 1–7 guttulate, measuring 1.1–3.1 μm in diam (Figure 4J). *Conidiogenous cell* compartments cylindrical, 1–7 guttulate, measuring (2.8 −)3.9–8.3(− 9.2) × (1.1 −)1.7–2.0(− 3.1) μm. *Conidia* hyaline, surface smooth, terminal or lateral in position, usually oval to spherical in shape, one-celled or in short chains of 2–9 cells, often branched, measuring (1.3−)1.6–2.7(− 3.1) × (2.5 −)2.5–4.8(− 6.2) μm (Figures 4K,L). *Chlamydospores* absent. Yeast-like cells absent. Sexual morph unknown.

*Culture characteristics*: The colony morphology on the PDA after 21 days was compact, dark gray in color, lobbed, with dark gray margin, striation emerging from the fissures extending to the center of the colony, surface villose, flat (Figure 4I). The colony grows slowly on PDA medium, reaching 15.5 mm in diam after four weeks at 25°C. The optimal growth temperature is 25°C. No growth was observed at 5, 30, and 35°C.

*Habitat*: A single isolate of this fungus was recovered from the medullary tissue of *Flavoparmelia caperata* collected in the Inner Mongolia Autonomous Region of China.

*Note*: *Cladophialophora flavoparmeliae* emerged as the basal diverging species of a clade that includes Taxon 1–7 described in this study along with many previously described species.

Taxon 9

***Cladophialophora moniliformis*** H.L. Si, R.L. Chang, T. Bose and X.X. Zheng sp. nov.

Figures 4M–P.

MycoBank no.: MB 846418.

*Etymology*: The name refers to the hyphae of this species that looks like a string of beads.

*Diagnosis*: *Cladophialophora moniliformis* is distinguished from closely related species by the production of ovate to spherical conidia.

*Type*: **China**: *Inner Mongolia Autonomous Region*, Chifeng city, Balin Right Banner, Mt. Qingyangcheng (44°14′2.86″ N, 118°41′35.92″ E), isolated from the medullary tissue of *Heterodermia pseudospeciosa* (SDNM418), 7 Jul. 2019, *H. L. Si*, 4181C = CGMCC3.20346 (The ex-holotype culture), dried culture HMAS 350276 (holotype specimen). GenBank Accession Numbers: ITS OP828642; LSU OP828618; SSU OP828602; TUB OP857257; and TEF OP831192.

*Description*: *Hyphae* hyaline to pale brown, smooth, straight, branched, septate, slightly constricted at the septa, compartments cylindrical, usually 0–2 guttulate, measuring 1.2–3.9 μm in diam (Figure 4N). *Conidiogenous cell* compartments cylindrical, 0–2 guttulate, measuring (2.5 −)2.9–8.5(− 12.4) × (1.2 −)2.0–2.2(− 2.5) μm. *Conidia* hyaline to light brown in color, smooth, thin-walled, lateral in position, usually globose to elliptic in shape, sometimes spherical, solitary or in short chains of 2–5 cells, branched, measuring (3.3 −)4.8–7.8(− 8.6) × (3.2 −)4.1–6.8(− 8.9) μm (Figures 4O,P). *Chlamydospores* absent. Yeast-like cells absent. Sexual morph unknown.

*Culture characteristics*: The colony morphology on the PDA after 21 days was compact, smoky gray in color with a diffusing dark gray margin, glossy around the margin, surface velutinous, flat, and margin entire with faint striations (Figure 4M). The colony grows slowly on PDA medium, reaching 27.5 mm in diam after four weeks at 25°C. The optimal growth temperature is 25°C. No growth was observed at 5, and 35°C.

*Habitat*: A isolate of this fungus was recovered from the medullary tissue of *Heterodermia pseudospeciosa* collected in the Inner Mongolia Autonomous Region of China.

*Note*: *Cladophialophora moniliformis* emerged as the sister taxon to a clade that includes *C. potulentorum* and *C. australiensis* with insignificant statistical support. *Cladophialophora moniliformis* also has substantial morphological differences with these two species. For example, the conidia of *C. moniliformis* are ovate to spherical in shape, whereas those of *C. potulentorum* and *C. australiensis* are ellipsoid (Crous et al., 2007). The conidia size of *C. moniliformis* is smaller than those of *C. potulentorum* and *C. australiensis* which is (6 −)8–10(− 13) × 2–3 μm and (7 −)8–12(− 15) × 3–4 μm, respectively.

## 4. Discussion

In this study, 15 *Cladophialophora* isolates were recovered from nine lichen species collected from Yunnan Province and the Inner Mongolia Autonomous Region of China. Analyses of morphological characteristics and molecular data using phylogenetic approaches revealed that these 15 isolates belonged to nine novel species from the genus. Consequently, these species were classified as *C. flavoparmeliae*, *C. guttulate*, *C. heterodermiae*, *C. holosericea*, *C. lichenis*, *C. moniliformis*, *C. mongoliae*, *C. olivacea*, and *C. yunnanensis*.

The phylogenetic analyses in this study were carried out using five gene regions, namely ITS, SSU, LSU, TUB, and TEF. For the majority of previously identified *Cladophialophora* species, only sequences for ITS and LSU are available. Sequences of SSU, TUB, and TEF were available for 23, 8, and 15 species, respectively. This is why the tree topologies of the single-gene and the concatenated datasets differed substantially. During the phylogenetic analyses, we also realized that ITS and LSU were sufficient for discriminating a majority of *Cladophialophora* species. This is most likely why several newly described species, such as *C. aquatica* (Boonmee et al., 2021), *C. cabanerensis* (Crous et al., 2020), and *C. tumbae* (Kiyuna et al., 2018), were based on these two gene regions. However, it was challenging for us to discriminate closely related species, such as *C. holosericea*, *C. olivacea*, and *C. guttulate*, without using sequences from protein-coding gene regions. For example, the ITS sequence was insufficient for distinguishing *C. olivacea* from *C. holosericea*. Similarly, LSU alone was unable to differentiate between *C. olivacea* and *C. guttulate*. TEF, on the other hand, was able to differentiate all three species. In the future, considering to amplify protein-coding gene regions, such as TEF and TUB, in addition to ITS and LSU would positively influence the taxonomy of *Cladophialophora*.

In the phylogeny, 46 species of *Cladophialophora* with available DNA sequences in GenBank were included. However, in the MycoBank, there are currently 54 species listed under this genus. Among the species excluded from this study, *C. brevicatenata* [MB 412793], *C. hachijoensis* [MB 412796], and *C. kellermaniana* [MB 412797], have been reclassified as *Tyrannosorus hanlinianus* (Shen et al., 2020), *Pseudocladosporium hachijoense* (Braun, 1998), and *Alternaria malorum* (Braun et al., 2003), respectively. We compared the micro-morphological characteristics of *C. cladoniae* [MB 800397], *C. hawksworthii* [MB 800398], *C. megalosporae* [MB 800399], and *C. normandinae* [MB 800400] with those of our nine species. These four *Cladophialophora* species have conidiomata (Diederich et al., 2013), a characteristic that was absent from our species. However, we were unable to compare the morphology of *C. bennettii* [MB 491905] to our species due to the unavailability of publication information for this species in any of the recognized databases, such as MycoBank and Index Fungorum. This evidence shows that all nine new species identified in this study were previously undocumented.

The species of *Cladophialophora* formed three distinct clades in the phylogenetic tree constructed using the concatenated dataset. Among these, *C. hostae*, *C. scillae*, and *C. behniae* formed the basal clade. Based on some previous studies, the phylogenetic position of this clade within *Cladophialophora* is controversial because these species might represent a new family (Gueidan et al., 2014; Quan et al., 2020). Similarly, the clade that included *C. proteae*, *C. eucalypti*, *C. pucciniophila*, *C. modesta*, *C. sylvestris*, *C. humicola*, and *C. minutissima* might not also include fungi from *Cladophialophora*. In a recent study, fungi from this clade grouped into various other families within Chaetothyriales (Quan et al., 2020). We opted to include these species in our phylogenetic studies because they are still classified as *Cladophialophora*. However, based on the data from this study and previous research, we propose a full taxonomic revision of the genus *Cladophialophora*.

A majority of *Cladophialophora* species, including those described in this study, emerged as a monophyletic clade with significant branch support values. This clade also included the type species, *C. carrionii*. Previously, based on SSU, LSU, and RPB1 sequence data, this clade included two phylogenetic groups: the Carrionii-clade and the Bantiana-clade (Badali et al., 2008; de Hoog et al., 2011). However, in a recent phylogeny of Chaetothyriales using ITS and LSU data, the existence of these clades was disputed (Quan et al., 2020). In this study, a phylogenetic tree constructed using a concatenated dataset showed the existence of the Carrionii-clade and the Bantiana-clade, but without significant branch support. Aside from that, the species composition of these clades did not completely overlap with that of Badali et al. (2008). This suggests that Carrionii-clade and Bantiana-clade are not well defined and should be used with caution.

Among the isolates of the species identified in this study, polymorphism was detected between sequences for the same gene region, for example, *C. lichenis* and *C. yunnanensis*. Sun et al. (2020) and Badali et al. (2009) while describing *C. nyingchiensis* and *C. carrionii*, respectively, also reported this trend. There are two possible explanations for this phenomenon. These fungi have multiple copies of the ITS and TUB genes, as do many other fungi (Fourie et al., 2014; Zhao et al., 2014). Alternatively, these sequence differences between the isolates might also be the product of cryptic species. This is because multiple copies of TEF in fungi have yet to be reported. However, TEF sequences varied by 20 bp among *C. nyingchiensis* isolates (Sun et al., 2020). This dilemma may be resolved in the future by species discovery and the recovery of additional isolates of identified species.

Lichenicolous fungi can exclusively parasitize the mycobiont (mycoparasites), the photobiont (phycoparasites), or sometimes both by forming haustoria (Lawrey and Diederich, 2003; Diederich et al., 2018). Irrespective of their host choice, lichenicolous fungi can be further classified into two types. These are slow-growing species that cause no or minor symptoms in their hosts, and fast-growing ones that are highly pathogenic to lichens (Harutyunyan et al., 2008). Based on these facts, we hypothesize that all nine *Cladophialophora* species described in this study were of the former type. The relationship between these nine species and their lichen host is either biotrophic or commensal (Lawrey and Diederich, 2003). This explains why we saw no symptoms on the lichen from which we isolated these fungi. However, infection trials are necessary to confirm their interaction with their hosts.

Results of this study indicated that *Cladophialophora* is an important lineage of lichenicolous fungi. However, a majority of lichen-associated species are yet to be discovered. We still do not know the precise role of these fungi and black fungi as a whole in lichen thallus. The lack of symptoms in the lichen thalli where these fungi were isolated suggests that these fungi can also be saprophytes or stress-associated latent pathogens that only exhibit symptoms when the host lichen is subjected to stress (Lücking et al., 2021). In addition, a large number of guttles in the hyphae of all *Cladophialophora* species identified in this study, especially *C. guttulate*. Therefore, there is scope for evaluating the potential to use these fungi in the production of microbial oil.

## Data availability statement

The datasets presented in this study can be found in online repositories. The names of the repository/repositories and accession number(s) can be found in the article/Supplementary material.

## Author contributions

RC and HS contributed to the conceptualization. YCW, XZ, and GZ performed the methodology and conducted the formal analysis. RC and TB was written the original draft preparation. YCW, YRW, YL, and SZ was performed the experiment. SL and MD carried out the resources. RC, HS, and TB wrote, reviewed, and edited the manuscript and directed the data. RC and GZ were responsible for project management and funding access. All authors contributed to the article and approved the submitted version.

## Funding

This study was supported by the Qingchuang Talents Induction Program of Shandong Higher Education Institution in 2021, Open Fund for Instruments and Equipment of Shandong Normal University, the “Startup Fund” awarded to Runlei Chang by the Shandong Normal University and National College Students Innovative Entrepreneurship Training Programs [202210445022].

## Conflict of interest

The authors declare that the research was conducted in the absence of any commercial or financial relationships that could be construed as a potential conflict of interest.

## Publisher’s note

All claims expressed in this article are solely those of the authors and do not necessarily represent those of their affiliated organizations, or those of the publisher, the editors and the reviewers. Any product that may be evaluated in this article, or claim that may be made by its manufacturer, is not guaranteed or endorsed by the publisher.

## Footnotes

^1^https://www.phylo.org

^2^http://tree.bio.ed.ac.uk/software/tracer

^3^https://imagej.nih.gov/ij/

## References

[ref1] Altschul S. F. Gish W. Miller W. Myers E. W. Lipman D. J. (1990). Basic local alignment search tool. J. Mol. Biol. 215, 403–410. doi: 10.1016/s0022-2836(05)80360-2 2231712

[ref2] Ametrano C. G. Muggia L. Grube M. (2019). “Extremotolerant black Fungi from rocks and lichens” in Fungi in extreme environments: Ecological role and biotechnological significance. eds. Tiquia-Arashiro S. M. Grube M. (Cham: Springer International Publishing)

[ref3] Badali H. Carvalho V. O. Vicente V. Attili-Angelis D. Kwiatkowski I. B. Van Den Ende A. H. G. G.. (2009). Cladophialophora saturnica sp. nov., a new opportunistic species of Chaetothyriales revealed using molecular data. Med. Mycol. 47, 51–62. doi: 10.1080/13693780802291452, PMID: 18720218

[ref4] Badali H. Gueidan C. Najafzadeh M. Bonifaz A. Van Den Ende A. G. De Hoog G. (2008). Biodiversity of the genus Cladophialophora. Stud. Mycol. 61, 175–191. doi: 10.3114/sim.2008.61.18, PMID: 19287540 PMC2610306

[ref5] Boonmee S. Wanasinghe D. N. Calabon M. S. Huanraluek N. Chandrasiri S. K. U.. (2021). Fungal diversity notes 1387–1511: taxonomic and phylogenetic contributions on genera and species of fungal taxa. Fung. Div. 111, 1–335. doi: 10.1007/s13225-021-00489-3, PMID: 34899100 PMC8648402

[ref6] Braun U. (1998). A monograph of Cercosporella, Ramularia and allied genera (phytopathogenic hyphomycetes). Additions to host range and distribution. Eching: IHW-Verlag Eching.

[ref7] Braun U. Crous P. Dugan F. Groenewald J. Z. Hoog S. (2003). Phylogeny and taxonomy of Cladosporium-like hyphomycetes, including Davidiella gen. Nov., the teleomorph of Cladosporium s. Mycol. Prog. 2, 3–18. doi: 10.1007/s11557-006-0039-2

[ref8] Brunauer G. Blaha J. Hager A. Turk R. Stocker-Worgotter E. Grube M. (2007). An isolated lichenicolous fungus forms lichenoid structures when co-cultured with various coccoid algae. Symbiosis 44, 127–136.

[ref9] Carbone I. Kohn L. M. (1999). A method for designing primer sets for speciation studies in filamentous ascomycetes. Mycologia 91, 553–556. doi: 10.1080/00275514.1999.12061051

[ref10] Crous P. W. Schubert K. Braun U. De Hoog G. S. Hocking A. D. Shin H. D.. (2007). Opportunistic, human-pathogenic species in the Herpotrichiellaceae are phenotypically similar to saprobic or phytopathogenic species in the Venturiaceae. Stud. Mycol. 58, 185–217. doi: 10.3114/sim.2007.58.07 18491000 PMC2104740

[ref11] Crous P. W. Wingfield M. J. Chooi Y. H. Gilchrist C. L. M. Lacey E. Pitt J. I.. (2020). Fungal planet description sheets: 1042-1111. Persoonia 44, 301–459. doi: 10.3767/persoonia.2020.44.11, PMID: 33116344 PMC7567971

[ref12] Crous P. W. Wingfield M. J. Guarro J. Cheewangkoon R. Van Der Bank M. Swart W. J.. (2013). Fungal planet description sheets: 154-213. Persoonia 31, 188–296. doi: 10.3767/003158513X675925, PMID: 24761043 PMC3904050

[ref13] Darriba D. Taboada G. L. Doallo R. Posada D. (2012). jModelTest 2: more models, new heuristics and parallel computing. Nat. Methods 9:772. doi: 10.1038/nmeth.2109, PMID: 22847109 PMC4594756

[ref14] De Hoog G. S. Vicente V. A. Najafzadeh M. J. Harrak M. J. Badali H. Seyedmousavi S. (2011). Waterborne Exophiala species causing disease in cold-blooded animals. Persoonia 27, 46–72. doi: 10.3767/003158511X614258, PMID: 22403476 PMC3251318

[ref15] Diederich P. (2010). Sclerococcum cladoniae, a new lichenicolous hyphomycete on Cladonia from Luxembourg. Bull. Soc. Nat. Luxemb. 20, 57–59. doi: 10.1002/chin.198925079

[ref16] Diederich P. Ertz D. Lawrey J. D. Sikaroodi M. Untereiner W. A. (2013). Molecular data place the hyphomycetous lichenicolous genus Sclerococcum close to Dactylospora (Eurotiomycetes) and S. parmeliae in Cladophialophora (Chaetothyriales). Fungal Divers. 58, 61–72. doi: 10.1007/s13225-012-0179-4

[ref17] Diederich P. Lawrey J. D. Ertz D. (2018). The 2018 classification and checklist of lichenicolous fungi, with 2000 non-lichenized, obligately lichenicolous taxa. Bryologist 121:386. doi: 10.1639/0007-2745-121.3.340

[ref18] Fourie A. Wingfield M. J. Wingfield B. D. Barnes I. (2014). Molecular markers delimit cryptic species in Ceratocystis sensu stricto. Mycol. Prog. 14:1020. doi: 10.1007/s11557-014-1020-0

[ref19] Gostinčar C. Muggia L. Grube M. (2012). Polyextremotolerant black fungi: oligotrophism, adaptive potential, and a link to lichen symbioses. Front. Microbiol. 3:e000390. doi: 10.3389/fmicb.2012.00390, PMID: 23162543 PMC3492852

[ref20] Groenewald J. Z. Nakashima C. Nishikawa J. Shin H. D. Park J. H. Jama A. N.. (2013). Species concepts in Cercospora: spotting the weeds among the roses. Stud. Mycol. 75, 115–170. doi: 10.3114/sim0012, PMID: 24014899 PMC3713887

[ref21] Gueidan C. Aptroot A. Da Silva Cáceres M. E. Badali H. Stenroos S. (2014). A reappraisal of orders and families within the subclass Chaetothyriomycetidae (Eurotiomycetes, Ascomycota). Mycol. Prog. 13:990. doi: 10.1007/s11557-014-0990-2

[ref22] Harutyunyan S. Muggia L. Grube M. (2008). Black fungi in lichens from seasonally arid habitats. Stud. Mycol. 61, 83–90. doi: 10.3114/sim.2008.61.08, PMID: 19287530 PMC2610299

[ref23] Hawksworth D. L. Mariette S. C. (2003). A first checklist of lichenicolous fungi from China. Jun Wu Xi Tong 22, 359–363.

[ref24] Katoh K. Standley D. M. (2013). MAFFT multiple sequence alignment software version 7: improvements in performance and usability. Mol. Biol. Evol. 30, 772–780. doi: 10.1093/molbev/mst010, PMID: 23329690 PMC3603318

[ref25] Khan E. Sohnle P. G. (2019). “Cutaneous fungal infections☆” in Encyclopedia of microbiology. ed. Schmidt T. M. (Oxford: Academic Press), 793–798.

[ref26] Kiyuna T. An K.-D. Kigawa R. Sano C. Sugiyama J. (2018). Two new Cladophialophora species, C. tumbae sp. nov. and *C. tumulicola* sp. nov., and chaetothyrialean fungi from biodeteriorated samples in the Takamatsuzuka and Kitora tumuli. Mycoscience 59, 75–84. doi: 10.1016/j.myc.2017.08.008

[ref27] Kondratyuk S. Y. Lőkös L. Halda J. P. Haji Moniri M. Farkas E. Park J. S.. (2016a). New and noteworthy lichen-forming and lichenicolous fungi 4. Acta Bot. Hung 58, 75–136. doi: 10.1556/034.58.2016.1-2.4

[ref28] Kondratyuk S. Y. Lőkös L. Halda J. P. Roux C. Upreti D. K. Schumm F.. (2017). New and noteworthy lichen-forming and lichenicolous fungi 6. Acta Bot. Hung 59, 137–260. doi: 10.1556/034.59.2017.1-2.7

[ref29] Kondratyuk S. Y. Lőkös L. Halda J. P. Upreti D. K. Mishra G. K. Haji Moniri M.. (2016b). New and noteworthy lichen-forming and lichenicolous fungi 5*. Acta Bot. Hung 58, 319–396. doi: 10.1556/abot.58.2016.3-4.7

[ref30] Kondratyuk S. Lőkös L. Tschabanenko S. Haji Moniri M. Farkas E. Wang X.. (2013). New and noteworthy lichen-forming and lichenicolous fungi. Acta Bot. Hung 55, 275–349. doi: 10.1556/abot.55.2013.3-4.9

[ref31] Kondratyuk S. Y. Upreti D. K. Mishra G. K. Nayaka S. Ingle K. K. Orlov O. O.. (2020). New and noteworthy lichen-forming and lichenicolous fungi 10. Acta Bot. Hung 62, 69–108. doi: 10.1556/034.62.2020.1-2.6

[ref32] Kumar S. Stecher G. Li M. Knyaz C. Tamura K. (2018). MEGA X: molecular evolutionary genetics analysis across computing platforms. Mol. Biol. Evol. 35, 1547–1549. doi: 10.1093/molbev/msy096, PMID: 29722887 PMC5967553

[ref33] Lawrey J. D. Diederich P. (2003). Lichenicolous Fungi: interactions, evolution, and biodiversity. Bryologist 106, 80–120. doi: 10.1639/0007-2745(2003)106[0080:LFIEAB]2.0.CO;2

[ref34] Lücking R. Leavitt S. D. Hawksworth D. L. (2021). Species in lichen-forming fungi: balancing between conceptual and practical considerations, and between phenotype and phylogenomics. Fungal Divers. 109, 99–154. doi: 10.1007/s13225-021-00477-7

[ref35] Miller M.A. Pfeiffer W. Schwartz T. (2011). The CIPRES science gateway: a community resource for phylogenetic analyses, in: Proceedings of the 2011 TeraGrid conference: extreme digital discovery, 1–8. doi: 10.1145/2016741.2016785

[ref36] Muggia L. Grube M. (2018). Fungal diversity in lichens: from extremotolerance to interactions with algae. Life 8:15. doi: 10.3390/life8020015, PMID: 29789469 PMC6027233

[ref37] Obase K. Douhan G. W. Matsuda Y. Smith M. E. (2016). Cladophialophora floridana and Cladophialophora tortuosa, new species isolated from sclerotia of Cenococcum geophilum in forest soils of Florida, USA. Mycoscience 57, 26–34. doi: 10.1016/j.myc.2015.07.005

[ref38] O'donnell K. Cigelnik E. (1997). Two divergent intragenomic rDNA ITS2 types within a monophyletic lineage of the fungus Fusarium are nonorthologous. Mol. Phylogenet. Evol. 7, 103–116. doi: 10.1006/mpev.1996.0376, PMID: 9007025

[ref39] Quan Y. Muggia L. Moreno L. F. Wang M. Al-Hatmi A. M. S. Da Silva Menezes N.. (2020). A re-evaluation of the Chaetothyriales using criteria of comparative biology. Fungal Divers. 103, 47–85. doi: 10.1007/s13225-020-00452-8

[ref40] Ronquist F. Teslenko M. Van Der Mark P. Ayres D. L. Darling A. Höhna S.. (2012). MrBayes 3.2: efficient Bayesian phylogenetic inference and model choice across a large model space. Syst. Biol. 61, 539–542. doi: 10.1093/sysbio/sys029, PMID: 22357727 PMC3329765

[ref41] Shen M. Zhang J. Q. Zhao L. L. Groenewald J. Z. Crous P. W. Zhang Y. (2020). Venturiales. Stud. Mycol. 96, 185–308. doi: 10.1016/j.simyco.2020.03.001, PMID: 32904190 PMC7452091

[ref42] Stamatakis A. (2014). RAxML version 8: a tool for phylogenetic analysis and post-analysis of large phylogenies. Bioinformatics 30, 1312–1313. doi: 10.1093/bioinformatics/btu033, PMID: 24451623 PMC3998144

[ref43] Sun W. Su L. Yang S. Sun J. Liu B. Fu R.. (2020). Unveiling the hidden diversity of rock-inhabiting fungi: Chaetothyriales from China. J Fungi 6:187. doi: 10.3390/jof6040187, PMID: 32987844 PMC7711927

[ref44] Sutton D. A. Rinaldi M. G. Sanche S. E. (2009). “Dematiaceous fungi” in Clinical mycology. eds. Anaissie E. J. Mcginnis M. R. Pfaller M. A.. 2nd ed (Edinburgh: Churchill Livingstone)

[ref45] Tang J. Zhuang K. Ran X. Dai Y. Ran Y. (2017). Chromoblastomycosis caused by *Cladophialophora carrionii*. Indian J. Dermatol. Venereol. Leprol. 93, 495–506. doi: 10.1590/abd1806-4841.20187321, PMID: 28540878

[ref46] Usui E. Takashima Y. Narisawa K. (2016). Cladophialophora inabaensis sp. nov., a new species among the dark septate endophytes from a secondary forest in Tottori Japan. Microbes Environ. 31, 357–360. doi: 10.1264/jsme2.ME16016, PMID: 27265343 PMC5017814

[ref47] Vilgalys R. Hester M. (1990). Rapid genetic identification and mapping of enzymatically amplified ribosomal DNA from several Cryptococcus species. J. Bacteriol. 172, 4238–4246. doi: 10.1128/jb.172.8.4238-4246.1990, PMID: 2376561 PMC213247

[ref48] White T. J. Bruns T. Lee S. Taylor J. W. (1990). “Amplification and direct sequencing of fungal ribosomal RNA genes for phylogenetics” in PCR protocols: a guide to methods and applications. eds. Innis M. A. Gelfand D. H. Sninsky J. J. White T. J. (San Diego (California): Academic Press), 315–322.

[ref49] Zhang Y.-J. Zhang S. Liu X. Wen H. A. Wang M. (2010). A simple method of genomic DNA extraction suitable for analysis of bulk fungal strains. Lett. Appl. Microbiol. 51, 114–118. doi: 10.1111/j.1472-765X.2010.02867.x, PMID: 20536704

[ref50] Zhao Z. Liu H. Luo Y. Zhou S. An L. Wang C.. (2014). Molecular evolution and functional divergence of tubulin superfamily in the fungal tree of life. Sci. Rep. 4:6746. doi: 10.1038/srep06746, PMID: 25339375 PMC5381371

[ref51] Zoller S. Scheidegger C. Sperisen C. (1999). PCR primers for the amplification of mitochondrial small subunit ribosomal DNA of lichen-forming ascomycetes. Lichenologist 31, 511–516. doi: 10.1006/lich.1999.0220

