# Supplementary figures and images for: Nine new species of black lichenicolous fungi from the genus Cladophialophora (Chaetothyriales) from two different climatic zones of China
Source: Front Microbiol. 2023 Jun 16;14:1191818. doi: 10.3389/fmicb.2023.1191818 (PMC10312087; doi:10.3389/fmicb.2023.1191818)

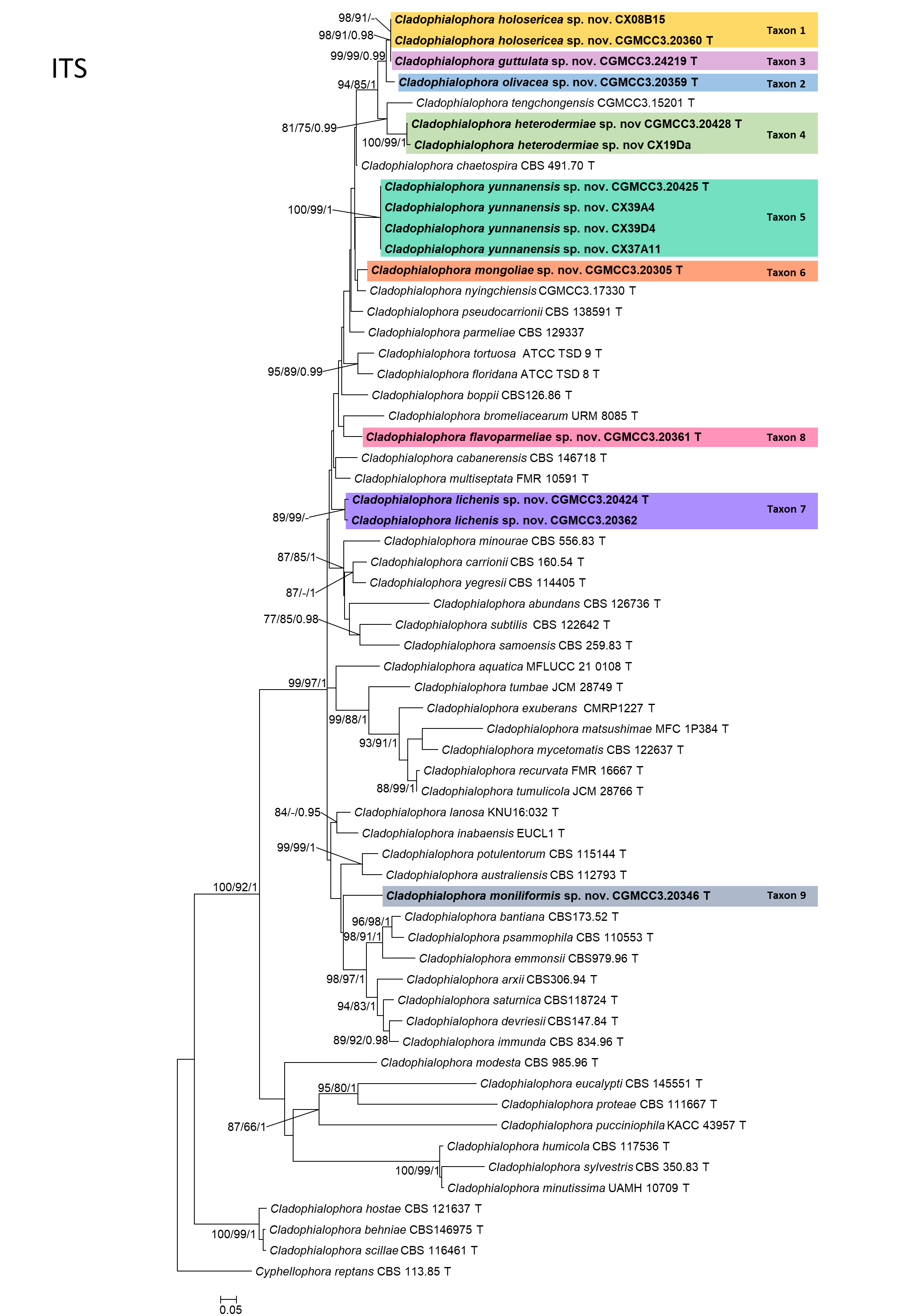

Supplement: Supplementary file 1 [file Image_1.TIF]

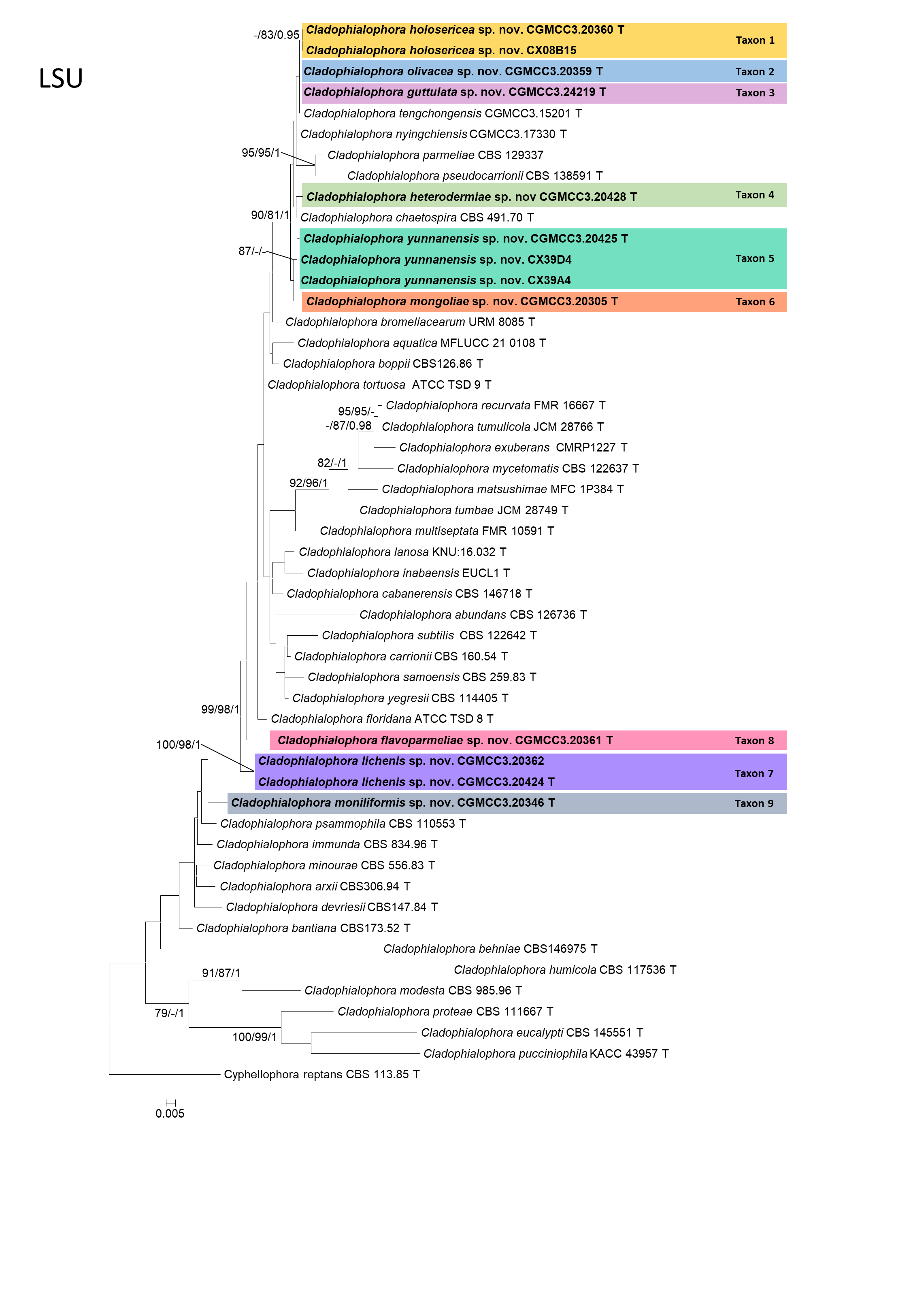

Supplement: Supplementary file 2 [file Image_2.TIF]

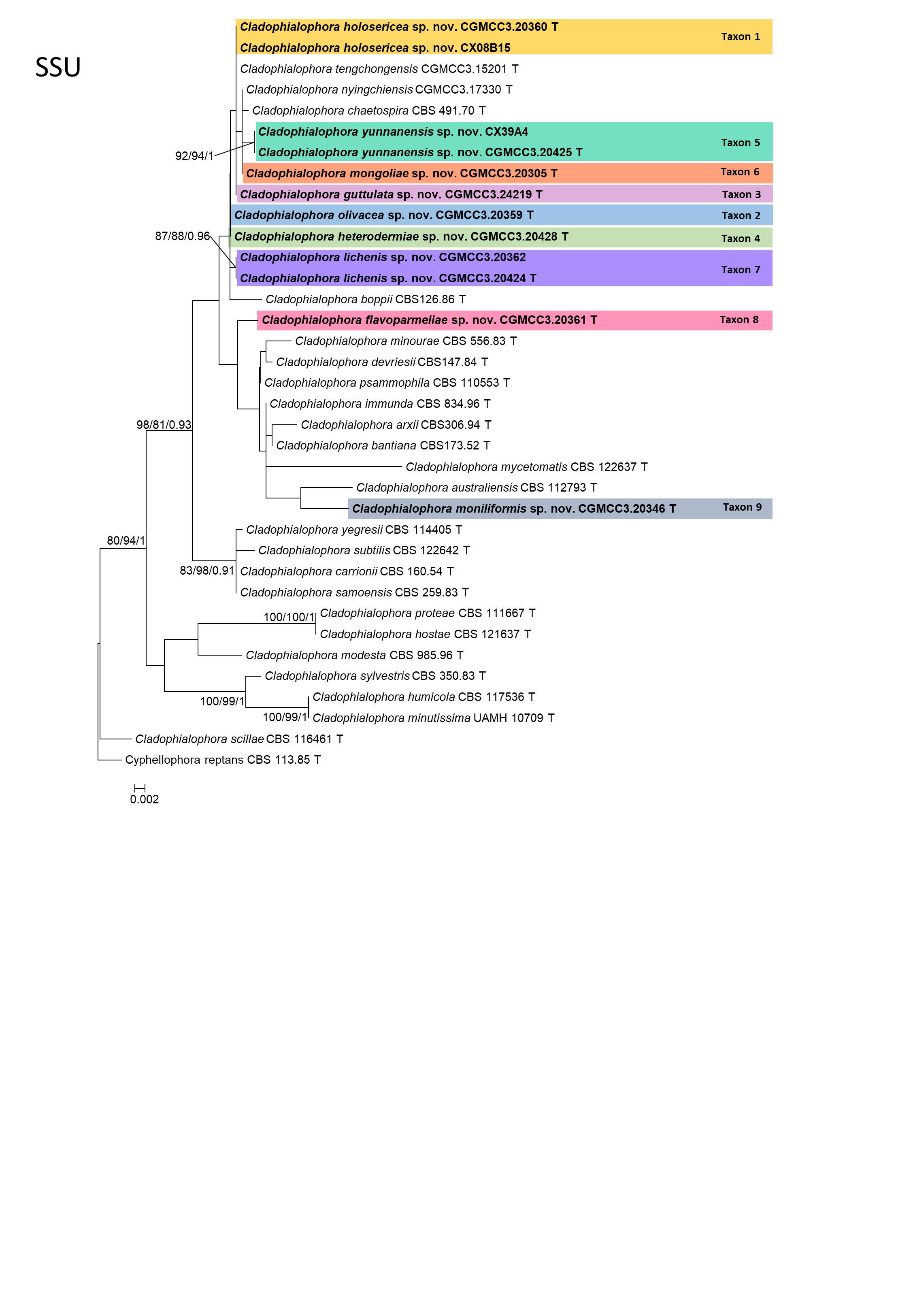

Supplement: Supplementary file 3 [file Image_3.TIF]

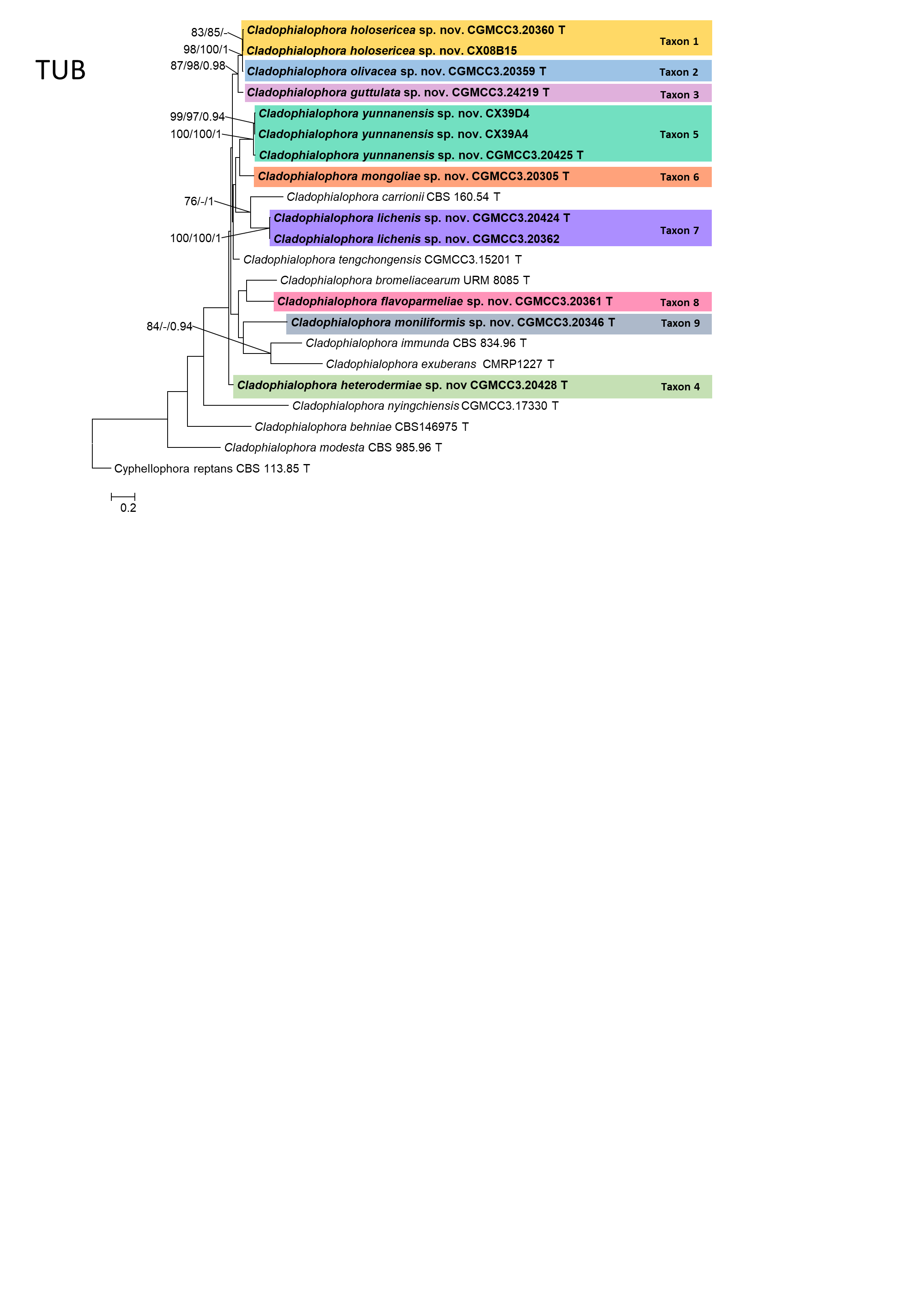

Supplement: Supplementary file 4 [file Image_4.TIF]

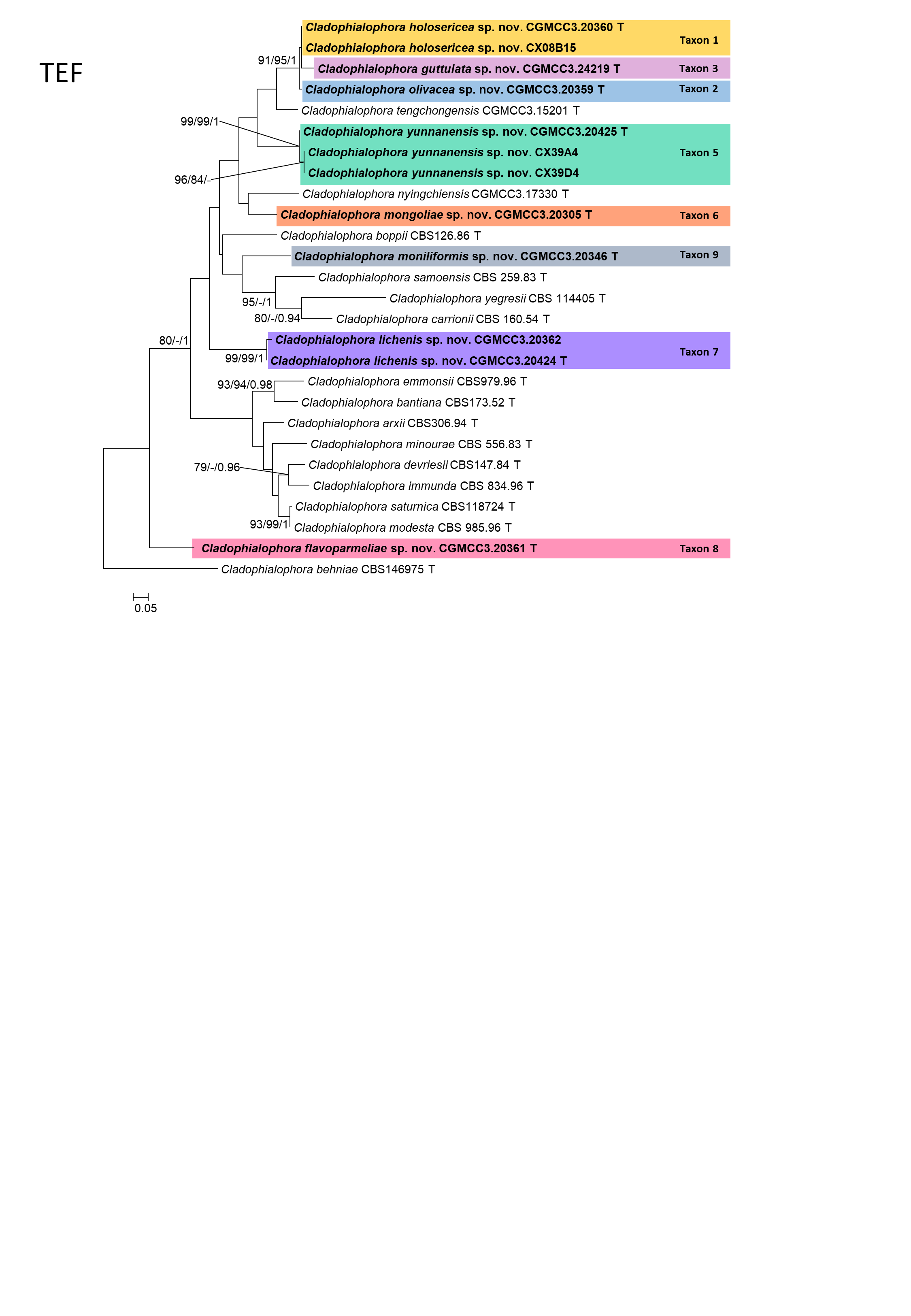

Supplement: Supplementary file 5 [file Image_5.TIF]
